# Zfp335 establishes eTreg lineage and neonatal immune tolerance by targeting Hadha-mediated fatty acid oxidation

**DOI:** 10.1172/JCI166628

**Published:** 2023-10-16

**Authors:** Xin Wang, Lina Sun, Biao Yang, Wenhua Li, Cangang Zhang, Xiaofeng Yang, Yae Sun, Xiaonan Shen, Yang Gao, Bomiao Ju, Yafeng Gao, Dan Liu, Jiapeng Song, Xiaoxuan Jia, Yanhong Su, Anjun Jiao, Haiyan Liu, Lianjun Zhang, Lan He, Lei Lei, WanJun Chen, Baojun Zhang

**Affiliations:** 1 Department of Pathogenic Microbiology and Immunology, School of Basic Medical Sciences, Xi’an Jiaotong University, Xi’an, Shaanxi, China.; 2 Institute of Infection and Immunity, Translational Medicine Institute, Xi’an Jiaotong University Health Science Center, Xi’an, Shaanxi, China.; 3 Key Laboratory of Environment and Genes Related to Diseases (Xi’an Jiaotong University), Ministry of Education, Xi’an, Shaanxi, China.; 4 Xi’an Key Laboratory of Immune-Related Diseases, Xi’an, Shannxi, China.; 5 Department of Otolaryngology Head and Neck Surgery, The First Affiliated Hospital of Xi’an Jiaotong University, Xi’an, Shaanxi, China.; 6 Department of Rheumatology and Immunology, The First Affiliated Hospital of Xi’an Jiaotong University, Xi’an, Shaanxi, China.; 7 Institute of Systems Medicine, Chinese Academy of Medical Sciences and Peking Union Medical College, Beijing, China.; 8 Suzhou Institute of Systems Medicine, Suzhou, China.; 9 Department of Rheumatology and Immunology, The First Affiliated Hospital of Xi’an Medical University, Xi’an, Shaanxi, China.; 10 Mucosal Immunology Section, National Institute of Dental and Craniofacial Research (NIDCR), NIH, Bethesda, Maryland, USA.

**Keywords:** Autoimmunity, Immunology, Adaptive immunity, Autoimmune diseases, T cells

## Abstract

Regulatory T cells (Tregs) are instrumental in maintaining immune tolerance and preventing destructive autoimmunity, but how heterogeneous Treg populations are established remains largely unknown. Here, we show that *Zfp335* deletion in Tregs failed to differentiate into effector Tregs (eTregs) and lose Treg-suppressive function and that KO mice exhibited early-onset lethal autoimmune inflammation with unrestricted activation of conventional T cells. Single-cell RNA-Seq analyses revealed that Zfp335-deficient Tregs lacked a eTreg population and showed dramatic accumulation of a dysfunctional Treg subset. Mechanistically, Zfp335-deficient Tregs displayed reduced oxidative phosphorylation and dysfunctional mitochondrial activity. Further studies revealed that Zfp335 controlled eTreg differentiation by regulating fatty acid oxidation (FAO) through direct targeting of the FAO enzyme Hadha. Importantly, we demonstrate a positive correlation between ZNF335 and HADHA expression in human eTregs. Our findings reveal that Zfp335 controls FAO-driven eTreg differentiation to establish immune tolerance.

## Graphical abstract

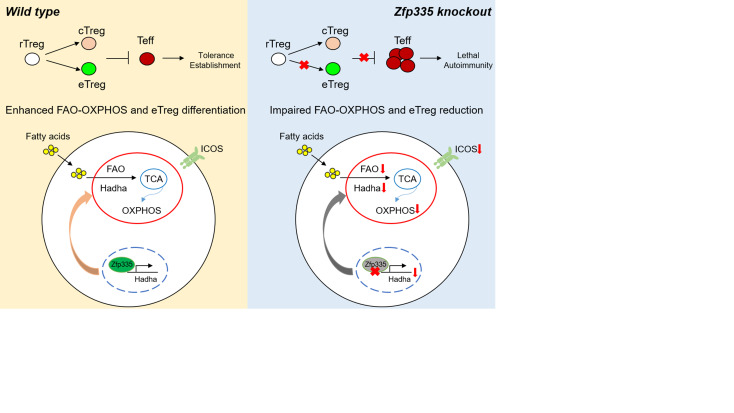

## Introduction

Regulatory T cells (Tregs) expressing forkhead box P3 (Foxp3) play a key role in the establishment and maintenance of peripheral immune tolerance through the prevention and suppression of autoimmunity (1). Defects in the number and function of Tregs lead to autoreactive T cell– and B cell–triggered responses (2–4). The inflammatory environment may cause Tregs to convert into pathogenic ex-Tregs characterized by the production of inflammatory cytokines including IFN-γ, IL-4, or IL-17 (5, 6). Consequently, this uncontrolled inflammation in multiple vital organs causes destructive and even fatal autoimmune diseases.

Natural Tregs are generated in the thymus (tTregs) and migrate into peripheral lymphoid organs and nonlymphoid tissues. After their homeostasis in the periphery, Tregs differentiate into heterogeneous populations including resting Tregs (rTregs) and activated effector Tregs (eTregs) (7–9). rTregs exhibit more quiescent features, as they express the naive T cell markers CD62L and CCR7, whereas eTregs highly express activated T cell markers (CD44, ICOS, KLRG1) (10) and inhibitory molecules (CTLA-4, GITR, IKZF4) and/or secrete higher levels of suppressive cytokines (TGF-β, IL-10, IL-35) (11, 12). Although a number of studies showed that severe loss of bulk Tregs results in a robust autoreactive immune response, there is increasing evidence suggesting a strong association between decreased numbers of eTregs and the occurrence of autoimmune diseases (13–17). Therefore, eTregs may serve as direct executors that exert regulatory functions to maintain immune homeostasis. Yet, it remains unclear how heterogeneous Treg populations are differentiated and controlled during their establishment and maintenance of immune tolerance.

Continuous TCR and IL-2 signaling has been demonstrated to be required for eTreg differentiation and function (8, 18–20). However, the differentiation of resting T cells into effector T cells requires, fundamentally, the metabolic support of sufficient energy production (21–23). For example, activation of PI3K/AKT/mTOR signaling enhances glycolytic metabolism and inhibits Foxp3 expression, which plays negative roles in the differentiation, survival, and function of Tregs (22, 24–29). However, following Treg activation, mTOR signaling is upregulated, which in turn induces lipid synthesis, mevalonate metabolism, and mitochondrial function and regulates eTreg homeostasis and function (25, 30–33). Accumulated evidence suggests that eTreg generation relies more on oxidative phosphorylation (OXPHOS) relative to glycolysis (31, 34). For example, liver kinase B1 (LKB1), a bioenergetic sensor, regulates OXPHOS-dependent metabolism to ensure the survival and functional fitness of Tregs (35, 36). Thus, metabolism may be a key determinant of the quiescence exit and effector specification of Tregs, and the molecular pathways controlling metabolic programs in this context remain unclear.

Zinc finger protein 335 (Zfp335), also known as the nuclear hormone receptor coregulator (NRC) interacting factor 1 (NIF-1) in humans, consists of a 13 C2H2 zinc finger–repeating structure consisting of 1,337 amino acids (37). The C2H2-ZF family plays important roles in the regulation of immune cell development, differentiation, and effector function (38–42). Zfp335 regulates gene transcription by interacting with H3K4 methyltransferase complexes and coactivators or by directly binding to gene promoters (37, 43–45). Germline knockout of *Zfp335* is embryonically lethal, whereas the *Zfp335bloto* allele, a missense mutation derived from *N*-ethyl-*N*-nitrosourea (ENU) mutagenesis, leads to a significant reduction in the number of peripheral T cells due to defects in the maturation and migration of thymocytes (46). Our previous studies also demonstrate a severe block at the double-negative (DN) stage of thymocyte development upon LckCre-induced Zfp335 deletion (47). Zfp335 is also involved in memory CD8^+^ T cell differentiation by its direct regulation of transcription factor 7 (TCF7) expression (47). Clearly, Zfp335 plays critical roles in multiple aspects of T cell biology, yet the role of Zfp335 in Tregs remains unknown.

Here, we show that Treg-specific deletion of Zfp335 caused uncontrolled systemic autoimmunity with multiorgan inflammation, leading to early death of mice. Zfp335 deficiency resulted in impaired Treg-immunosuppressive function, together with a profound reduction of Tregs in the inflammatory environment. Strikingly, comprehensive single-cell transcriptomics analysis revealed that Zfp335 deficiency led to a loss of eTregs with accumulation of a dysfunctional Treg (dysTreg) subpopulation. Mechanistically, Zfp335 regulates fatty acid oxidation (FAO) by direct targeting of the FAO enzyme Hadha, which in turn controls eTreg lineage differentiation. Therefore, our data uncover the essential role of Zfp335 in controlling FAO-driven eTreg lineage commitment and establishing neonatal immune tolerance.

## Results

### Zfp335 in Tregs is indispensable for establishing neonatal immune tolerance.

**Figure 1:**
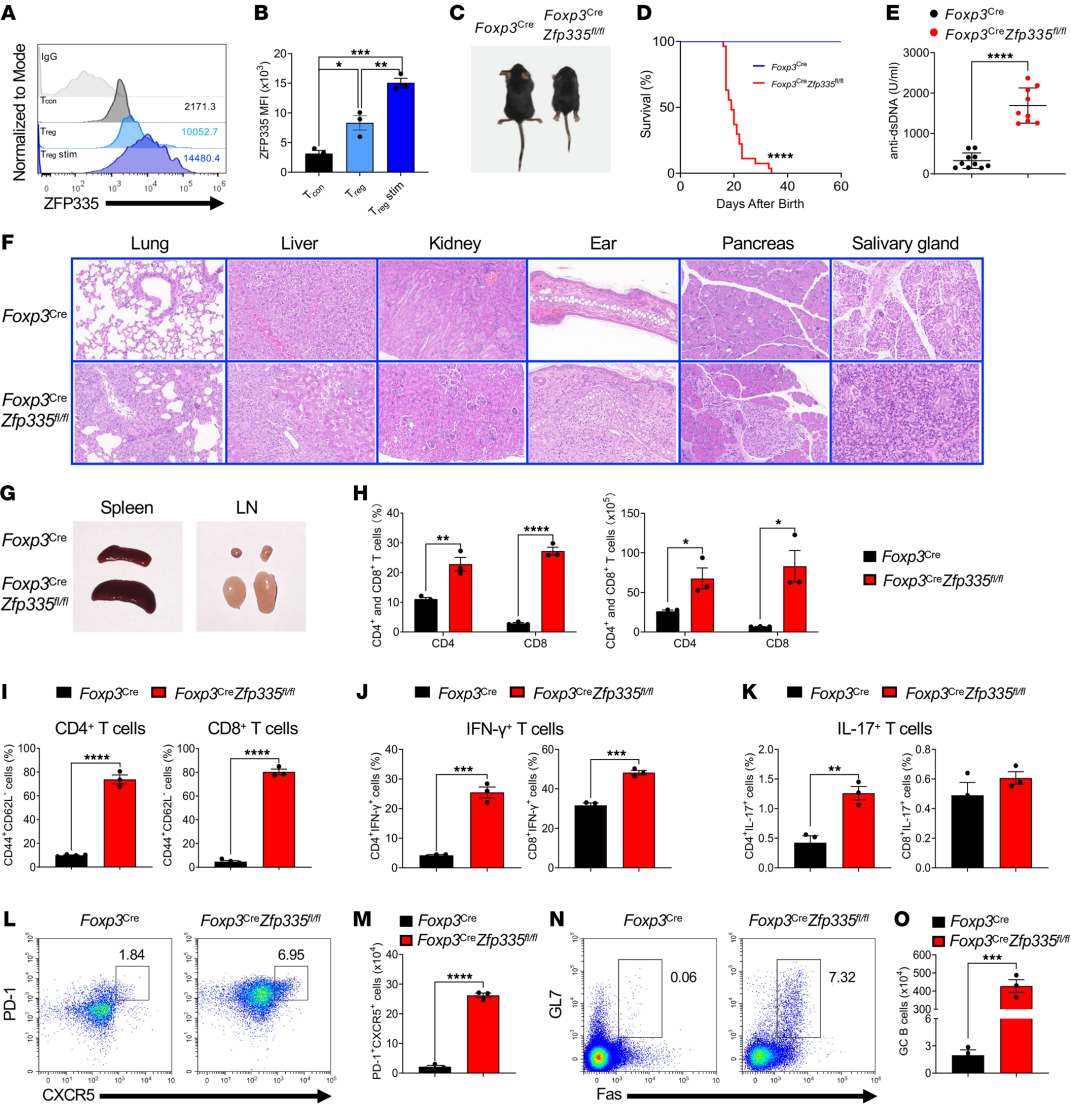
Spontaneous multiorgan immunopathology after Treg-specific ablation of Zfp335. (**A**) ZFP335 expression in naive CD4^+^ T cells (Tcons), Tregs, and Tregs stimulated (stim) with anti-CD3/anti-CD28 Abs and IL-2 for 2 days. (**B**) MFI of ZFP335 in **A**. (**C**) Representative images of 3-week-old WT (*Foxp3^Cre^*) and KO (*Foxp3^Cre^* *Zfp335^fl/fl^*) mice. (**D**) Survival curves for WT and KO mice (*n* = 27). (**E**) ELISA quantification of dsDNA-specific IgG in serum of WT (*n* = 10) and KO (*n* = 9) mice. (**F**) H&E staining of sections of the indicated organs from WT and KO mice (original magnification, ×20). (**G**) Representative images of spleens and peripheral LNs from WT and KO mice. (**H**) Frequencies and numbers of splenic CD4^+^ and CD8^+^ T cells from WT and KO mice (*n* = 3). (**I**–**K**) Frequencies of CD44^+^CD62L^–^ cells (**I**), IFN-γ^+^ cells (**J**), and IL-17^+^ (**K**) in splenic CD4^+^ and CD8^+^ T cells (*n* = 3–4). (**L**) Representative FACS plots of PD-1 and CXCR5 expression in splenic CD4^+^ T cells. (**M**) Number of PD-1^+^CXCR5^+^ cells in splenic CD4^+^ T cells (*n* = 3). (**N**) Representative FACS plots of GL7 and Fas expression in splenic CD19^+^ B cells. (**O**) Number of GL7^+^Fas^+^ GC B cells (*n* = 3). Data are representative of 3 independent experiments and are shown as the mean ± SEM. *P ≤ 0.05, **P ≤ 0.01, ***P ≤ 0.001, and ****P ≤ 0.0001, by 1-way ANOVA with Tukey’s multiple-comparison test (**B**), log-rank (Mantel-Cox) test (**D**), and 2-sided, unpaired *t* test (**E**, **H**–**K**, **M**, and **O**).

To determine whether *Zfp335* plays a role in Tregs, we first assessed ZFP335 expression in conventional T cells (Tcons) and Tregs. Flow cytometric analysis showed that Tregs had increased ZFP335 expression compared with Tcons (Figure 1, A and B). ZFP335 expression levels were further increased in Tregs upon stimulation with anti-CD3 and anti-CD28 Abs, together with IL-2 (Figure 1, A and B), whereas no change was observed in Tcons after T cell receptor (TCR) and IL-2 stimulation (Supplemental Figure 1; supplemental material available online with this article; https://doi.org/10.1172/JCI166628DS1). The results suggest that *Zfp335* might be important for Treg lineage differentiation and/or suppressive function.

To address this, we crossed *Zfp335*-floxed mice with *Foxp3^Cre^* mice (48) to generate mice with Treg-specific deletion of Zfp335 (designated *Foxp3^Cre^* *Zfp335^fl/fl^* mice, also referred to herein as KO mice) (Supplemental Figure 2A). With Zfp335 deficiency in Tregs (Supplemental Figure 2B), *Foxp3^Cre^* *Zfp335^fl/fl^* mice had a markedly smaller body size in their early life (Figure 1C), and most of them died within approximately 3–4 weeks (Figure 1D). We detected markedly elevated amounts of anti-dsDNA Abs in the serum of *Foxp3^Cre^* *Zfp335^fl/fl^* mice (Figure 1E), indicating an occurrence of autoimmunity. Histopathologic analysis revealed that, compared with *Foxp3^Cre^* mice (designated as WT mice), *Foxp3^Cre^* *Zfp335^fl/fl^* mice spontaneously developed multiorgan immunopathology with disrupted tissue structure, subcutaneous hyperplasia, and massive inflammatory infiltrates (Figure 1F and Supplemental Figure 3). We also observed enlarged spleens and peripheral lymph nodes (LNs) in *Foxp3^Cre^* *Zfp335^fl/fl^* mice (Figure 1G). The frequencies of CD8^+^ T cells were significantly increased in the spleen, peripheral LNs, and mesenteric LNs (mLNs) in *Foxp3^Cre^* *Zfp335^fl/fl^* mice, whereas CD4^+^ T cells were only significantly increased in the spleen (Figure 1H and Supplemental Figure 4, A–C). In contrast, the thymus size of *Foxp3^Cre^* *Zfp335^fl/fl^* mice was markedly reduced, accompanied by severe impairment of both thymic cortical and medullary compartments (Supplemental Figure 4D). The cell numbers of double-positive (DP) thymocytes and CD4^+^ and CD8^+^ single-positive T cells were also significantly decreased in *Foxp3^Cre^* *Zfp335^fl/fl^* mice (Supplemental Figure 4, E–G), which was most likely caused by overwhelming systemic inflammation (49).

We next assessed the activation status of peripheral T cells in 3-week-old mice. Compared with WT mice, *Foxp3^Cre^* *Zfp335^fl/fl^* mice showed significantly higher frequencies of CD44^+^CD62L^–^ activated or memory-like CD4^+^ and CD8^+^ T cells in the spleen (Figure 1I), LNs, and mLNs (Supplemental Figure 5, A–F). Moreover, both CD4^+^ and CD8^+^ T cells from *Foxp3^Cre^* *Zfp335^fl/fl^* mice produced large amounts of IFN-γ, while IL-17 expression was hardly detectable, as expected (50, 51) (Figure 1, J and K, and Supplemental Figure 5, G–L). Follicular helper CD4^+^ T (Tfh) cells expressing programmed cell death 1 (PD-1) and CXCR5 are specialized effector T cells that promote B cell functions and effective humoral responses (52, 53). We noticed that both the frequencies and absolute numbers of Tfh cells in the spleen and LNs were significantly increased in *Foxp3^Cre^* *Zfp335^fl/fl^* mice (Figure 1, L and M, and Supplemental Figure 6, A and B), with a corresponding expansion of germinal center (GC) B cells denoted by the expression of the GC signature markers GL7 and Fas (also known as CD95) (Figure 1, N and O, and Supplemental Figure 6, C and D). Additionally, Ly6C^+^ myeloid cells, a population of pathogenic cells produced during inflammation (54), were also elevated in the spleen and LNs (Supplemental Figure 7). Taken together, Zfp335 in Tregs were essential in establishing neonatal immune tolerance and preventing the development of autoimmune disease.

### Deletion of Zfp335 causes loss of Treg identity along with progression of inflammation.

**Figure 2:**
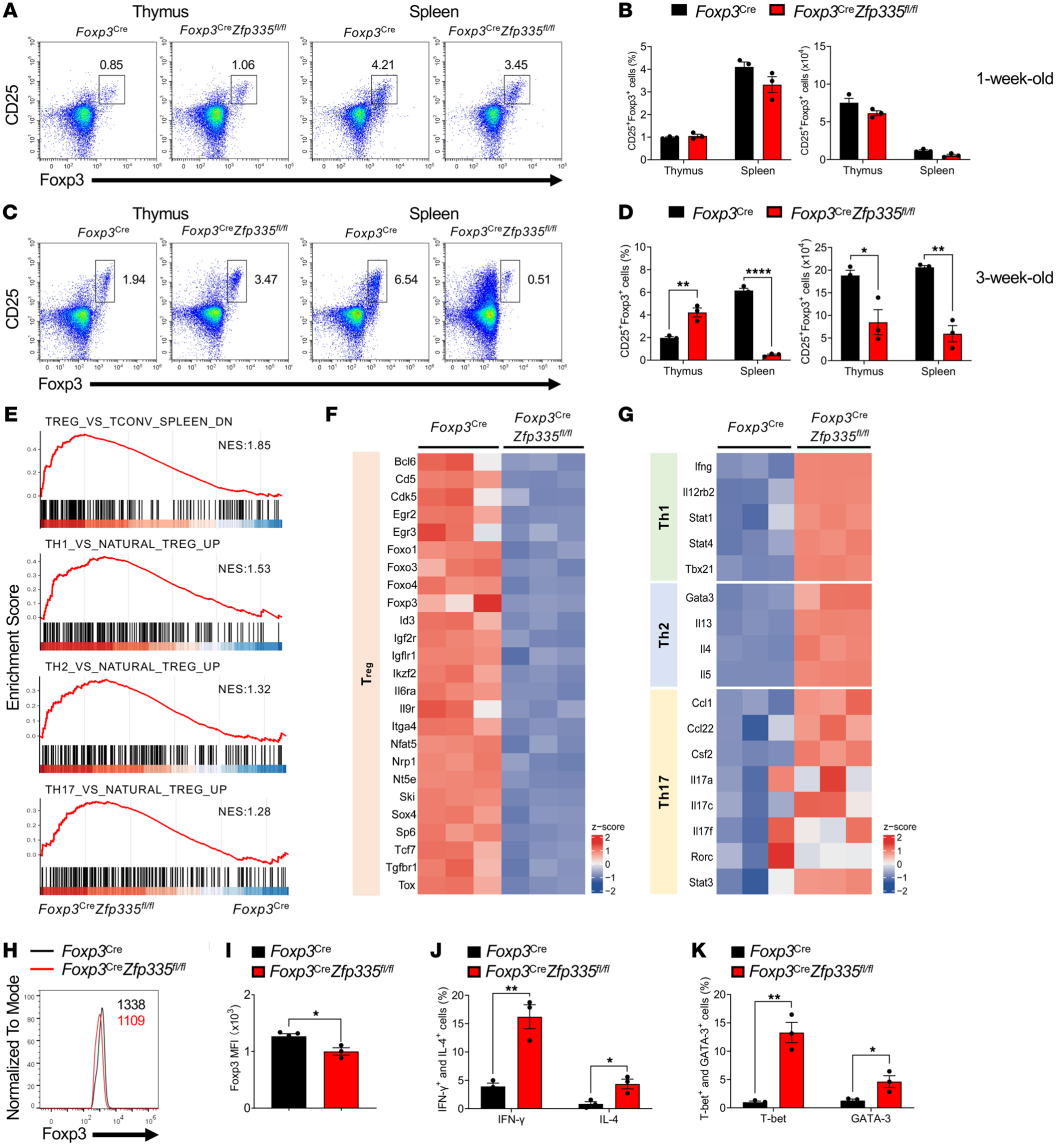
Impaired Treg signatures in Zfp335-deficient mice. (**A** and **B**) Representative FACS plots (**A**) and frequencies (**B**) of CD25^+^Foxp3^+^ Tregs in the thymus and spleen of 1-week-old WT and KO mice (*n* = 3). (**C** and **D**) Representative FACS plots (**C**) and frequencies (**D**) of CD25^+^Foxp3^+^ Tregs in the thymus and spleen of 3-week-old WT and KO mice (*n* = 3). (**E**) GSEA plots depict the identified WT and KO Treg gene sets associated with Th cell signatures. NES, normalized enrichment score. (**F** and **G**) Heatmaps of the DEGs associated with Treg (**F**) and Th1, Th2, and Th17 cell (**G**) differentiation between WT and KO groups. Heatmap colors represent the *z* score values relative to the control. (**H** and **I**) Representative FACS plots of Foxp3 expression (**H**) and MFI of Foxp3 (**I**) in Tregs from 3-week-old WT and KO mice (*n* = 3–4). (**J**) Frequencies of IFN-γ^+^ and IL-4^+^ Tregs in WT and KO mice (*n* = 3). (**K**) Frequencies of T-bet^+^ and GATA-3^+^ Tregs in WT and KO mice (*n* = 3). Data are representative of 3 independent experiments and are shown as the mean ± SEM. *P ≤ 0.05, **P ≤ 0.01, and ****P ≤ 0.0001, by 2-sided, unpaired *t* test.

Since there was an obvious syndrome of autoimmunity in the *Foxp3^Cre^* *Zfp335^fl/fl^* mice, we next investigated the direct effects of Zfp335 deficiency on Treg identity and population size. The percentages and numbers of Tregs (CD25^+^Foxp3^+^) in the thymus and spleen were comparable between 1-week-old *Foxp3^Cre^* *Zfp335^fl/fl^* mice and WT mice (Figure 2, A and B), suggesting that Zfp335 deletion had no effect on Treg development. However, compared with WT counterparts, the frequency and number of Tregs (CD25^+^Foxp3^+^) from 3-week-old *Foxp3^Cre^* *Zfp335^fl/fl^* mice were significantly decreased in the spleen (Figure 2, C and D). The absolute number of Tregs in the thymus was also significantly reduced, although the frequency was increased compared with WT tTregs, which was due to the decrease in DP thymocytes (Figure 2, C and D, and Supplemental Figure 4, D–G). The results were also confirmed by CD25 expression and the yellow fluorescent protein (YFP) marker (Supplemental Figure 8, A–F).

To better understand Treg properties in *Foxp3^Cre^* *Zfp335^fl/fl^* mice, we sorted Tregs from 2-week-old WT and KO mice and performed genome-wide transcriptomics analysis (RNA-Seq) and found that 3,072 genes were differentially expressed (>1.5-fold, *P* < 0.05) with 1,580 genes upregulated; 1,492 genes downregulated; and 11,253 genes unchanged (Supplemental Figure 9). We further performed gene set enrichment analysis (GSEA) on 2 sets of sorted Treg RNA-Seq data (KO vs. WT) (Figure 2E). Genes upregulated in Tcons compared with Tregs were highly enriched in the Zfp335-KO group. Meanwhile, the expression of Tcon (Th1, Th2, and Th17) signature genes was also enriched in the KO group, indicating a loss of identity in Zfp335-deficient Tregs. Additionally, the most significantly enriched transcription signatures associated with Th cell differentiation were summarized as heatmaps (Figure 2, F and G). Consistently, we found that prominent genes associated with the Treg signature were remarkably downregulated in Zfp335-deficient Tregs, including *Egr2*, *Foxo1*, *Foxo3*, *Foxp3*, *Ikzf2*, *Nrp1*, *Nt5e*, and *Tgfbr1*. The expression of genes associated with the Th1 signature (*Ifng*, *Il12rb2*, *Stat1*, *Stat4*, and *Tbx21*) and Th2 (*Gata3*, *Il13*, *Il4*, and *Il5*) was highly elevated in the KO group, whereas upregulation of the genes in the Th17 signature showed a similar but not completely consistent trend. A comprehensive list of all the gene hits is included in Supplemental Table 1.

The above findings prompted us to further investigate whether Zfp335 modulates Foxp3 protein expression. We found that Foxp3 expression was slightly decreased in 3-week-old KO (*Foxp3^Cre^* *Zfp335^fl/fl^*) Tregs (Figure 2, H and I). Kinetics studies revealed no changes in Foxp3 expression in 1- and 2-week-old mice but slightly reduced expression in 3-week-old mice after Zfp335 deficiency in all secondary lymphoid tissues (Supplemental Figure 10, A and B), indicating an unlikely direct regulation of Foxp3 expression by Zfp335. However, we further verified the elevated expression of Th1 (IFN-γ and T-bet) and Th2 (IL-4 and GATA-3) signature genes at the protein level in Tregs from the spleens of *Foxp3^Cre^* *Zfp335^fl/fl^* mice using FACS analysis (Figure 2, J and K).

However, when we assessed the changes in Treg and other Th cell signature genes in heterozygous *Foxp3^Cre/+^* *Zfp335^fl/fl^* female mice without autoreactive inflammation, we found that most of them were almost equally expressed in WT and Zfp335-deficient Tregs (Supplemental Figure 11).

Therefore, these data show that Zfp335-deficient Tregs lost their signatures while acquiring proinflammatory and dysfunctional features in mice, which was due to a secondary effect caused by inflammation rather than an intrinsic effect.

### Zfp335 is required for Treg-suppressive function in vitro and in vivo.

**Figure 3:**
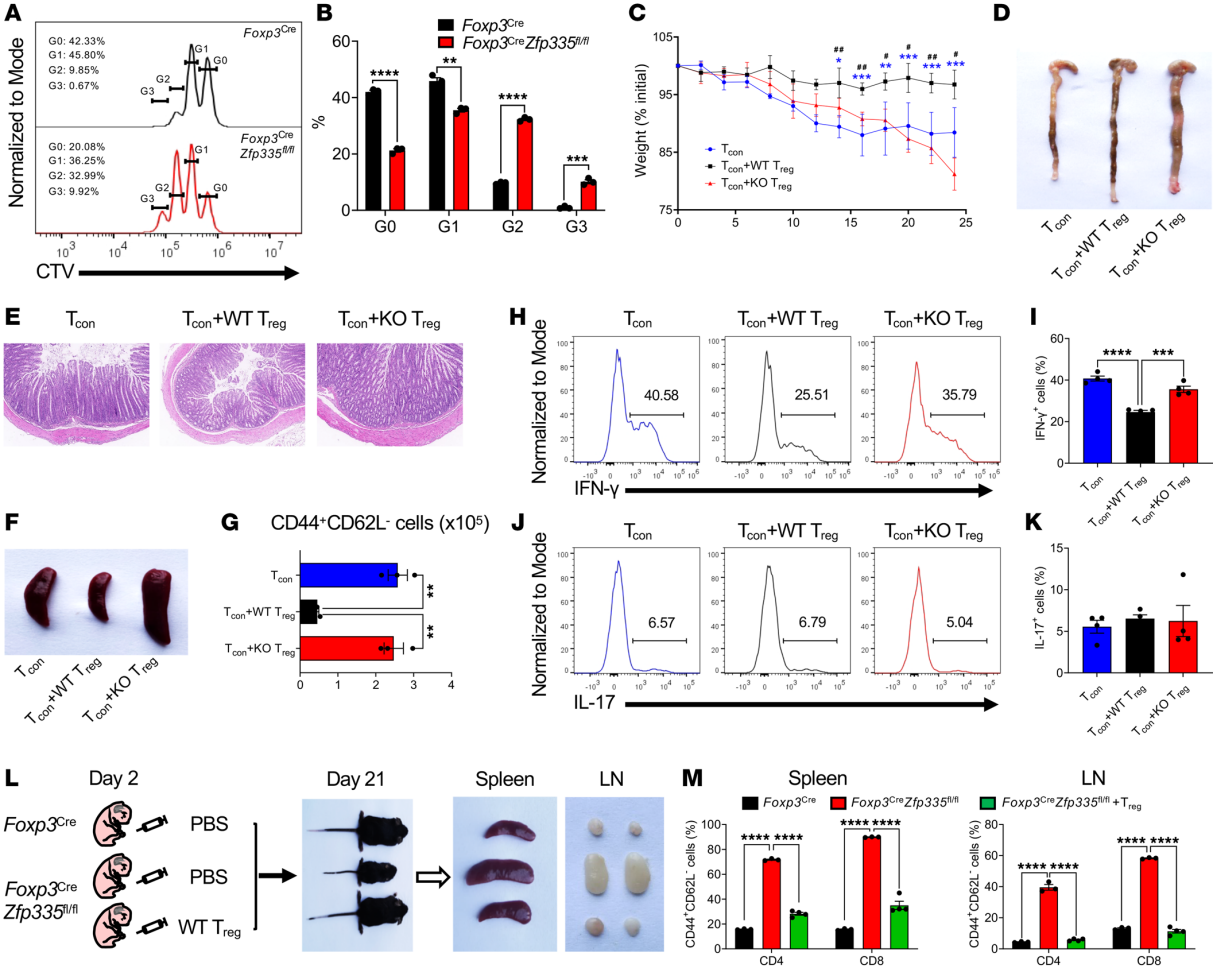
Zfp335 deficiency impairs the suppressive function of Tregs. (**A**) Representative histogram of CTV dilution of naive CD4^+^ T cells stimulated with anti-mCD3 Ab and antigen-presenting cells (APCs) in the presence of WT or KO Tregs for 60 hours. (**B**) Frequencies of each cell division (*n* = 4). (**C**) Frequencies of initial body weights of recipient mice 25 days after transfer with 5 × 10^5^ Tcons alone, or together with 2.5 × 10^5^ WT (Tcon+WT Treg) or KO (Tcon+KO Treg) Tregs. *P ≤ 0.05, **P ≤ 0.01, ***P ≤ 0.001 (Tcon+WT Treg vs. Tcon+KO Treg); ^#^*P* ≤ 0.05 and ^##^*P* ≤ 0.01 (Tcon vs. Tcon+WT Treg). (**D**–**F**) Representative images of colons (**D**), histology images of colon sections (**E**), and images of spleens (**F**) from recipient mice (original magnification, ×8). (**G**) Numbers of CD44^+^CD62L^–^ splenic CD4^+^ T cells (*n* = 3). (**H** and **I**) Representative FACS plots of IFN-γ expression (**H**) and frequencies of IFN-γ^+^ (**I**) in splenic CD4^+^ T cells. (**J** and **K**) Representative FACS plots of IL-17 expression (**J**) and frequencies of IL-17^+^ (**K**) in splenic CD4^+^ T cells (*n* = 4). (**L**) Schematic diagram of Treg transfer assay and images of recipient mice and spleens and LNs 19 days after the transfer. (**M**) Frequencies of CD44^+^CD62L^–^ cells in CD4^+^ and CD8^+^ T cells in spleens (left) and LNs (right) from recipient mice (*n* = 3–4). Data are representative of 2 independent experiments and are shown as the mean ± SEM. *P ≤ 0.05, **P ≤ 0.01, ***P ≤ 0.001, and ****P ≤ 0.0001; ^#^*P* ≤ 0.05 and ^##^*P* ≤ 0.01. Statistical significance was determined by 2-sided, unpaired *t* test (**B** and **C**) and 1-way ANOVA with Tukey’s multiple-comparison test (**G**, **I**, **K**, and **M**).

Immunosuppressive function of Tregs is essential for maintaining immune homeostasis and self-tolerance to prevent autoimmune disease. Therefore, we next investigated the role of Zfp335 in the suppressive function of Tregs by measuring their ability to suppress Tcons. Compared with WT counterparts, we observed that Zfp335-deficient Tregs from 10-day-old mice had a significantly impaired ability to suppress T cell proliferation when cocultured in vitro with naive T cells following TCR stimulation (55) (Figure 3, A and B). Consistently, we confirmed the impaired suppressive function of Tregs from heterozygous *Foxp3^Cre/+^* *Zfp335^fl/fl^* mice (Supplemental Figure 12). To explore the in vivo function of Tregs, we used a well-characterized T cell transfer model of colitis (56). The adoptive transfer of WT Tcons (CD4^+^CD25^–^CD45RB^hi^) into immunocompromised mice (recombinant activating gene 2–deficient [RAG2-deficient] mice) induced severe colitis, manifested by a gradual loss of body weight (Figure 3C), a shortened and thickened colon (Figure 3D), as well as bowel hyperplasia, disruption of epithelial cells, and inflammatory infiltrates (Figure 3E). The cotransfer of WT Tregs together with Tcons effectively suppressed the autoimmune symptoms, however, cotransfer of Zfp335-deficient Tregs failed to do so (Figure 3, C–E), suggesting an attenuation of the immunosuppressive capability of Zfp335-deficient Tregs. Moreover, the transfer of Tcons elicited overt autoimmunity and a larger spleen size (Figure 3F), a greater frequency of activated/effector T cells (Figure 3G), as well as augmented expression of the proinflammatory cytokine IFN-γ (Figure 3, H–K, and Supplemental Figure 13). Consistently, WT, but not Zfp335-deficient, Tregs prevented these pathological phenotypes (Figure 3, F–K, and Supplemental Figure 13).

Next, to determine whether normal Tregs are sufficient to prevent the autoimmune phenotypes in KO (*Foxp3^Cre^* *Zfp335^fl/fl^*) mice, we injected WT Tregs into 2-day-old KO pups and analyzed them after 19 days (Figure 3L). Strikingly, we observed that neonatal transfer of WT Tregs prevented all autoimmune phenotypes in the KO mice, including reduced body size and weight, enlarged spleen and LNs, inflammatory colitis phenotypes, and elevated expression of CD4^+^ and CD8^+^ T cells as well as of their effector proportions in the spleen, LNs, and mLNs (Figure 3, L and M, and Supplemental Figure 14). Importantly, when examining KO Tregs before and after WT Treg injection, we found that WT Treg treatment also restored altered Treg frequencies in the thymus (Supplemental Figure 15, A and B), whereas the percentage of KO Tregs in the spleen did not fully return to WT levels despite an increase to some extent (Supplemental Figure 15, C and D). The data collectively indicate that Zfp335 was indispensable for Treg-suppressive function.

### Zfp335 determines the effector versus the dysfunctional Treg fate.

**Figure 4:**
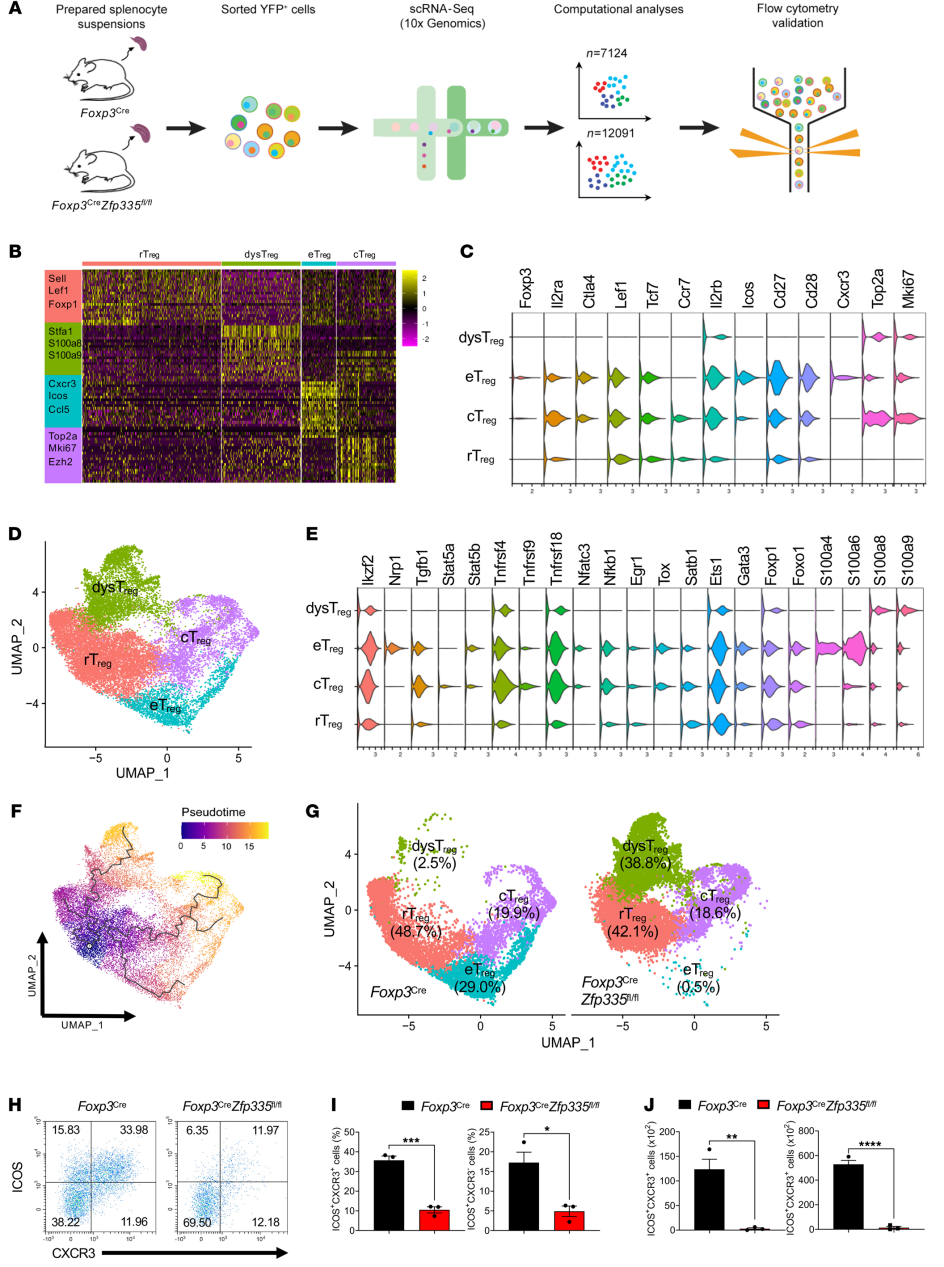
Single-cell transcriptomics delineates distinct Treg populations. (**A**) Schematic diagram of the experimental design, scRNA-Seq, data analysis, and validation. Tregs from 7-day-old WT and KO mice were sorted and subjected to scRNA-Seq by 10x Genomics. (**B**) Heatmap showing the relative expression of marker genes across different immune cell types. (**C**) Violin plots showing the Treg marker genes in each Treg cluster. (**D**) UMAP projections of Treg clusters, color-coded by cluster. (**E**) Violin plots showing the Treg signature genes specifically expressed in each Treg cluster. (**F**) Pseudotime plot shows the progression of 4 Treg populations reconstructed by monocle2 using scRNA-Seq data. (**G**) UMAP projections and percentage of Treg clusters in WT and KO mice. (**H**) Representative FACS plots of ICOS and CXCR3 expression in splenic Tregs from 8-day-old WT and KO mice. (**I** and **J**) Frequencies (**I**) and numbers (**J**) of ICOS^+^CXCR3^+^ (left) and ICOS^+^CXCR3^–^ (right) splenic Tregs (*n* = 3). Gene expression in **B**, **C**, and **E** is represented as the expression of normalized log_2_ (count +1). Data are representative of 3 independent experiments and are shown as the mean ± SEM (**H**–**J**). *P ≤ 0.05, **P ≤ 0.01, ***P ≤ 0.001, and ****P ≤ 0.0001, by 2-sided, unpaired *t* tests (**I** and **J**).

To determine the underlying mechanisms for Zfp335-mediated regulation of Treg identity and function, we performed single-cell transcriptomics (scRNA-Seq) analysis of YFP^+^ Tregs sorted from 7-day-old WT and KO (*Foxp3^Cre^* *Zfp335^fl/fl^*) mice using the 10x Genomics platform (Figure 4A). Potential doublets (WT = 726; KO = 1,451) were removed using the DoubletFinder (version 2.0) package at default settings (Supplemental Figure 16A) (57), followed by scRNA-Seq data normalization, after which no batch effects were detected by principal component analysis (PCA) analysis (Supplemental Figure 16B). After quality control and filtering, we obtained single-cell transcriptomes from 7,124 cells for WT mice and 12,091 cells for KO mice with similar median gene numbers (see Methods and Supplemental Figure 16C). Analysis of the top 20 differentially expressed genes (DEGs) across those cells revealed 4 cell clusters with unique transcriptional features (Figure 4B). A full list of the genes is included in Supplemental Table 2. Based on an unbiased integrative analysis across all WT and KO cells, these 4 clusters of Treg populations were annotated: rTregs, central Tregs (cTregs), eTregs, and dysTregs. The most salient markers of each Treg subset are presented in violin plots (Figure 4C), and the cell clustering is depicted using uniform manifold approximation and projection (UMAP) (Figure 4D) (58–60). Prominent genes highlighted in those populations revealed that rTregs had the lowest expression among most of the Treg marker genes, whereas cTregs and eTregs shared expression profiles similar to the high expression profiles of genes critical for Treg lineage differentiation and suppressive function (*Il2ra*, *Il2rb*, *Ctla4*, *Cd27*, *Cd28*, *Ikzf2*, *Tgfb1*, *Stat5b*, *Tnfrsf4*, *Tnfrsf9*, *Tnfrsf18*, and *Tox*), with *Ccr7*, *Top2a*, and *Mki67* more highly enriched in cTregs, while *Nrp1*, *Icos*, *Cxcr3*, *S100a4* and *S100a6* more highly enriched in eTregs (Figure 4, C–E). Interestingly, although dysTregs represented a cell population whose transcriptomes had the greatest similarities to rTreg transcriptomes, some genes, such as *Il2ra*, *Lef1*, *Tcf7*, *Cd27*, *Cd28*, *Tgfb1*, etc., were expressed at even lower levels in dysTregs. Of note, dysTreg expressed the proliferative markers *Top2a* and *Mki67* and had high expression levels of *S100a8* and *S100a9*, which are constitutively expressed in neutrophils and monocytes and involved in modulating the inflammatory response (61).

To further understand the function of Zfp335 in the differentiation path among Treg populations, we constructed differentiation trajectory analysis for those 4 Treg subsets via Monocle (version 3) pseudotime analysis (see Methods and Figure 4F). rTregs were located at the root of the trajectory tree, while cTregs, eTregs, and dysTregs were distributed along the trajectory in sequential order. The distribution of cell clusters captured by scRNA-Seq was compared side by side between WT and Zfp335-KO mice (Figure 4G). Treg populations in WT mice constituted the majority of cTregs (48.7%), eTregs (29%), rTregs (19.9%), and some dysTregs (2.5%). Zfp335 deficiency did not affect cTregs (42.1%) or rTregs (18.6%), at least in terms of their frequencies (Figure 4G). However, the eTreg population was almost completely lost in Zfp335-KO mice (0.5%), but we observed a striking accumulation of dysTregs (38.8%) (Figure 4G). Thus, it is likely that in physiological conditions, rTregs had mainly differentiated into eTregs, whereas Zfp335 deficiency may have driven rTregs into the dysTreg population. Consistently, we found that the frequencies of ICOS^+^CXCR3^+^ or ICOS^+^CXCR3^–^ eTregs were significantly decreased in 8-day-old *Foxp3^Cre^* *Zfp335^fl/fl^* mice compared with WT mice (Figure 4, H–J).

Furthermore, we analyzed the subpopulation changes in heterozygous *Foxp3*^Cre/+^ *Zfp335^fl/fl^* mice. The scRNA-Seq data showed that inducible T cell costimulator (ICOS) was specifically expressed in eTregs and that OX40 (also known as Tnfrsf4) was highly expressed in cTregs, but was expressed at lower levels in rTregs. We defined ICOS^–^OX40^lo^, ICOS^–^OX40^hi^, and ICOS^+^ cells as rTregs, cTregs, and eTregs, respectively. We found that only Zfp335-deficient ICOS^+^ eTregs were reduced in 1-week-old heterozygous *Foxp3*^Cre/+^ *Zfp335^fl/fl^* mice, while ICOS^–^OX40^lo^ rTregs and ICOS^–^OX40^hi^ cTregs had no significant changes (Supplemental Figure 17, A and B). Moreover, we examined other markers associated with eTregs. Zfp335-deficient CXCR3^+^ and Nrp1^+^ eTregs were consistently decreased in 1-week-old heterozygous *Foxp3*^Cre/+^ *Zfp335^fl/fl^* mice (Supplemental Figure 18, A and B). Meanwhile, we also observed a significant reduction of ICOS^+^CXCR3^+^ eTregs in other tissues (liver and lung) from these mice (Supplemental Figure 18, C and D).

Thus, the data reveal that Zfp335 regulated the proportions of distinct Treg subsets and that Zfp335 deficiency resulted in the accumulation of dysTregs at the expense of eTregs.

### Zfp335 controls eTreg differentiation through metabolic pathways.

**Figure 5:**
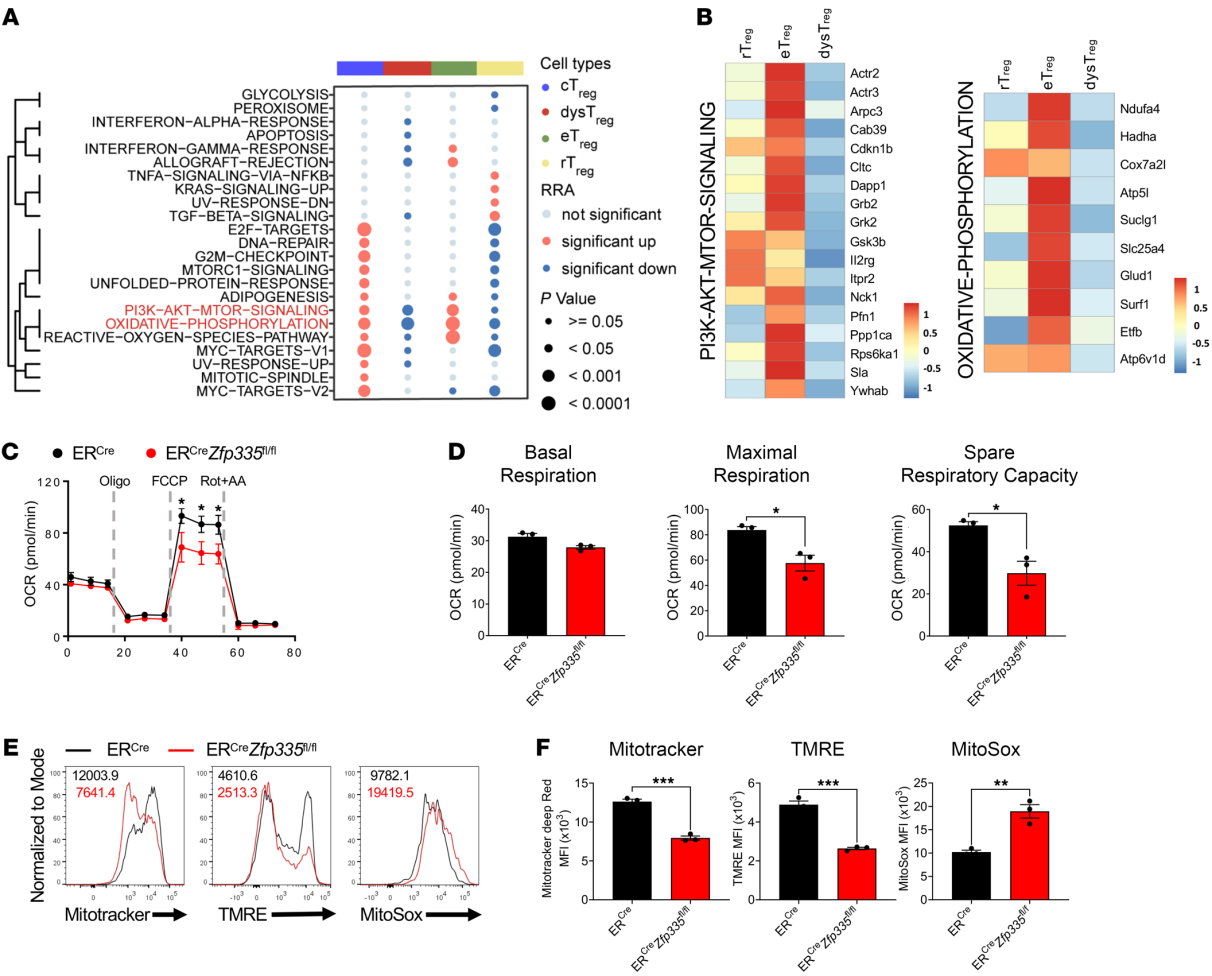
Zfp335 targets metabolism pathways of Tregs. (**A**) Hallmark pathways significantly enriched in rTreg, dysTregs, and eTregs based on RRA scores (see Methods). (**B**) Heatmap shows representative gene expression in PI3K/AKT/MTOR signaling and OXPHOS pathways. (**C**) CD4^+^CD25^+^CD44^–^ICOS^–^ rTregs sorted from ER*^Cre^* and ER*^Cre^* *Zfp335^fl/fl^* mice were activated with anti-mCD3/anti-CD28 Abs and IL-2. After a 12-hour stimulation, Seahorse analysis of the OCR in these cells was performed. Oligomycin (Oligo), FCCP, and rotenone plus antimycin A (Rot+AA) were injected as indicated. (**D**) Quantification of basal respiration, maximal respiration, and spare respiratory capacity in **C** (*n* = 3). (**E**) Representative FACS plots of MitoTracker Deep Red, TMRE, and MitoSox staining in ER*^Cre^* and ER*^Cre^* *Zfp335^fl/fl^* rTregs activated with anti-mCD3/anti-CD28 Abs and IL-2 for 5 days. (**F**) MitoTracker Deep Red, TMRE, and MitoSox MFI in **E** (*n* = 3). Data are representative of 2 or 3 independent experiments and are shown as the mean ± the SEM. *P ≤ 0.05, **P ≤ 0.01, and ***P ≤ 0.001, by 2-sided, unpaired *t* test.

It has been well appreciated that metabolic pathways are essential for T cell maintenance and function (62, 63). Given the dramatic imbalance between eTregs and dysTregs due to Zfp335 deficiency, we focused on those cell subsets. Notably, PI3K/Akt/mTOR and OXPHOS pathways had significantly higher rank aggregation algorithm (RRA) scores in the eTreg population but lower RRA scores in the dysTreg population (Figure 5A). Furthermore, 4 algorithms used to score gene sets confirmed our conclusion that eTregs had the most enriched PI3K/Akt/mTOR and OXPHOS signaling compared with dysTregs (Supplemental Figure 19, A and B). The upregulated genes (eTregs vs. dysTregs) were intersected with genes in the PI3K/Akt/mTOR and OXPHOS pathways, and 18 (Figure 5B and Supplemental Figure 19C) and 10 (Figure 5B and Supplemental Figure 19D) genes were screened out, respectively. We then performed Seahorse analysis to determine the oxygen consumption rate (OCR) as a measurement of OXPHOS and found that Zfp335 deletion led to a reduced OCR, especially with regard to maximal respiration and spare respiratory capacity (Figure 5, C and D). Given that OXPHOS takes place in the mitochondria in eukaryotic cells (64), we measured the mitochondrial mass and mitochondrial membrane potential of Zfp335-deficient Tregs (65). rTregs from ER*^Cre^* and ER*^Cre^* *Zfp335^fl/fl^* mice treated with tamoxifen were stimulated with anti-CD3/anti-CD28 Abs and IL-2 for 5 days before measurement of mitochondrial mass with MitoTracker staining, mitochondrial membrane potential with tetramethylrhodamine ethyl ester (TMRE) staining, and mitochondrial ROS (mitoROS) production by MitoSOX. As expected, Zfp335-deficient Tregs showed decreased MitoTracker staining and TMRE staining, but increased mitoROS, indicating a dysfunction of mitochondrial activity in the activated Tregs following Zfp335 deficiency (Figure 5, E and F). Furthermore, we also observed decreased mitochondrial mass in activated Tregs from heterozygous *Foxp3*^Cre/+^ *Zfp335^fl/fl^* mice (Supplemental Figure 20).

### Zfp335 controls eTreg differentiation by targeting the FAO enzyme Hadha.

**Figure 6:**
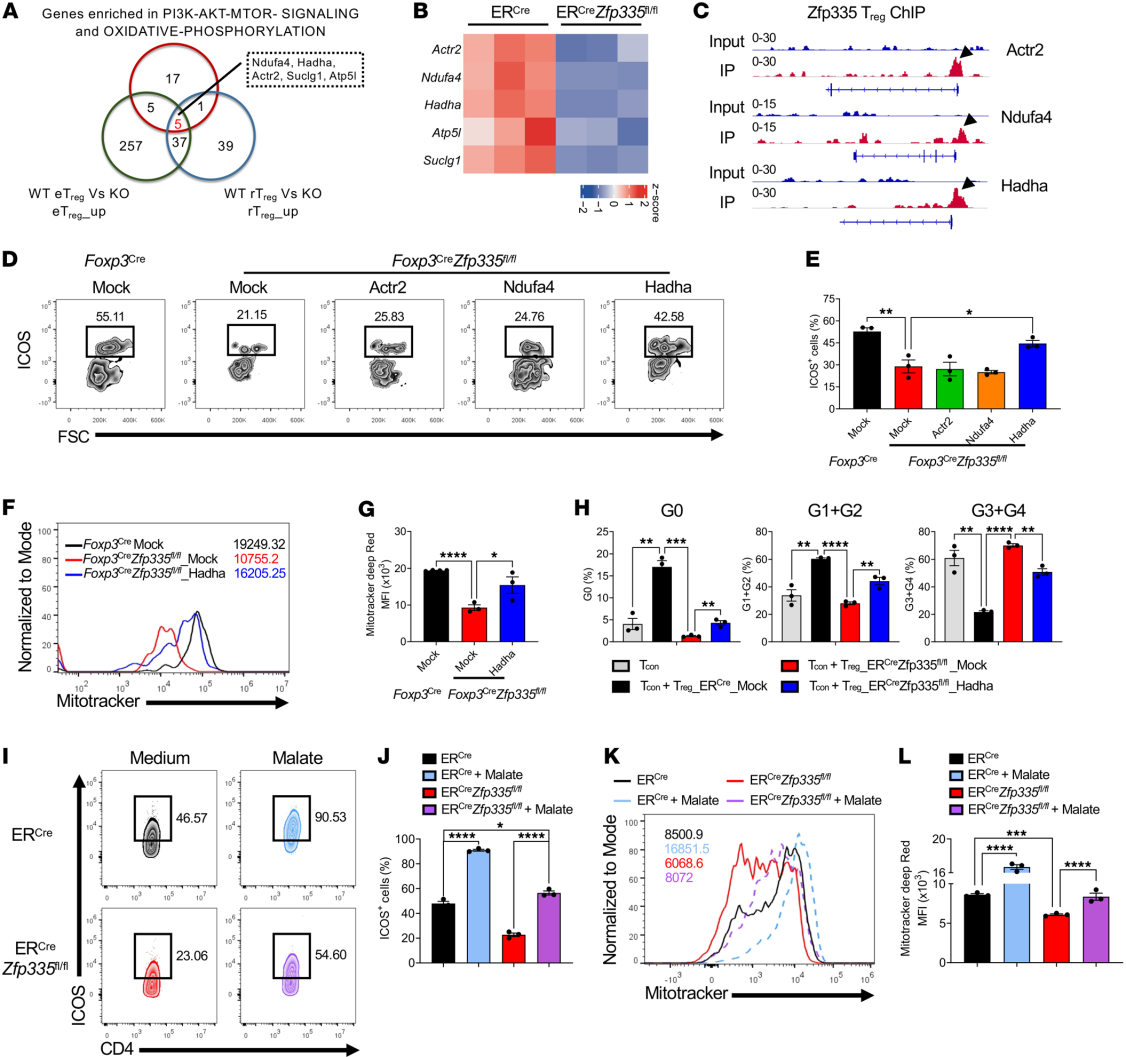
Zfp335 facilitates eTreg differentiation via direct targeting of Hadha. (**A**) Venn diagram shows the genes shared among the upregulated genes in eTregs and rTregs (WT vs. KO) and genes associated with metabolic pathways. (**B**) Heatmaps of the indicated genes between ER*^Cre^* and ER*^Cre^* *Zfp335^fl/fl^* Tregs. (**C**) ChIP-Seq peaks of Zfp335-bound regions in the indicated genes in WT Tregs compared with input. (**D** and **E**) CD4^+^YFP^+^CD44^–^ICOS^–^ Tregs were isolated from 8-day-old WT and KO mice. WT Tregs were transfected with mock, while KO Tregs were transfected with mock, *Ndufa4*, *Hadha*, and *Actr2* for 4 days. Representative FACS plots (**D**) and frequencies (**E**) of ICOS^+^ Tregs in different groups (*n* = 3). (**F** and **G**) Representative FACS plots (**F**) and MFI of MitoTracker Deep Red (**G**) in the indicated groups (*n* = 3). (**H**) Frequencies of each cell division of Tcons alone or in the presence of WT eTregs transfected with mock and KO eTregs transfected with mock or *Hadha*. (**I**–**L**) CD4^+^CD25^+^CD44^–^ICOS^–^ rTregs sorted from ER*^Cre^* and ER*^Cre^* *Zfp335^fl/fl^* mice were activated by anti-CD3/anti-CD28 Abs and IL-2 in the presence or absence of 30 mM malate. Two days later, the cells were collected and subjected to FACS analysis. (**I**) Representative FACS plots of ICOS^+^ Tregs in different groups. (**J**) Frequencies of ICOS^+^ Tregs (*n* = 3). (**K** and **L**) Representative FACS plots (**K**) and MFI of MitoTracker Deep Red (**L**) (*n* = 3). Data are representative of 2 or 3 independent experiments and are shown as the mean ± SEM. *P ≤ 0.05, **P ≤ 0.01, ***P ≤ 0.001, and ****P ≤ 0.0001, by 1-way ANOVA with Tukey’s multiple-comparison test.

We next explored the molecular mechanisms of Zfp335 regulation of eTreg differentiation. We hypothesized that the expression of target genes should begin to downregulate in rTregs, further decrease in eTregs after Zfp335 deletion, and be highly enriched in metabolic pathways. Therefore, we compared gene expression in eTregs and rTregs between WT and KO (*Foxp3^Cre^* *Zfp335^fl/fl^*) mice (Supplemental Tables 3 and 4), and further overlaid our findings with genes enriched in metabolic pathways in eTregs (Figure 6A). After comparing those 3 gene sets, we selected 5 overlapping genes (*Ndufa4*, *Hadha*, *Actr2*, *Suclg1*, and *Atp5l*) for further analysis. Downregulation of those genes was further confirmed in Zfp335-deficient Tregs from ER*^Cre^* *Zfp335^fl/fl^* mice treated with tamoxifen and from heterozygous *Foxp3*^Cre/+^ *Zfp335^fl/fl^* mice (Figure 6B and Supplemental Figure 21). To identify candidate genes directly targeted by Zfp335, we performed ChIP-Seq assay in WT Tregs and found that Zfp335 was bound to the promoter regions of 3 genes (*Actr2*, *Ndufa4*, and *Hadha*) (Figure 6C). Actin-related protein 2 (Actr2) is well known for functioning as a regulator of the actin network and thus the migration of diverse cell types (66). By forming a complex with Actr3, Actr2 participates in T cell homeostasis by maintaining surface TCR levels, probably through remodeling of the actin cytoskeleton (67, 68). Ndufa4, a subunit of complex IV of the mitochondrial respiratory chain, has been reported to promote cell proliferation and reduce apoptosis through activation of the OXPHOS pathway (69). Hadha is a FAO enzyme, and its mutation attenuates FAO (70). To further valid the direct regulation of these genes in eTreg differentiation, we overexpressed *Actr2*, *Ndufa4*, and *Hadha* genes separately in Zfp335-deficient rTregs. The results demonstrated that overexpression of *Hadha*, but not *Actr2* or *Ndufa4*, could rescue the reduced frequency of ICOS^+^ eTregs resulting from Zfp335 deficiency (Figure 6, D and E).

Consistently, *Hadha* overexpression also significantly rescued the reduced frequency of CXCR3^+^ and Nrp1^+^ eTregs caused by Zfp335 deficiency (Supplemental Figure 22, A and B). However, *Hadha* overexpression did not affect ICOS^–^OX40^lo^ rTregs or ICOS^–^OX40^hi^ cTregs (Supplemental Figure 22, C and D). Given these findings, we performed a luciferase assay to further confirm a direct binding of Zfp335 and the *Hadha* promoter region and obtained consistent ChIP-Seq results (Supplemental Figure 23). In addition, *Hadha* overexpression could also restore the decrease in MitoTracker expression in Zfp335-deficient Tregs (Figure 6, F and G). To confirm the effect of *Hadha* overexpression on the suppressive function of Zfp335-deficient Tregs, we cocultured CellTrace Violet–labeled (CTV-labeled) CD4^+^ naive T cells with eTregs or other Tregs infected with Mock or Hadha and found that *Hadha* overexpression restored the impaired suppressive function of Zfp335-deficient eTregs (Figure 6H), but not of other Tregs (Supplemental Figure 24). Thus, we propose that Zfp335 promoted eTreg differentiation and function via direct regulation of *Hadha* expression.

As Hadha is a key enzyme in FAO and sequentially regulates the citric acid (TCA) cycle (71), we sought to investigate the effect of malate, a potent intermediate in the TCA cycle, on eTreg differentiation. rTregs from ER*^Cre^* and ER*^Cre^* *Zfp335^fl/fl^* mice treated with tamoxifen were stimulated with anti-CD3/anti-CD28 Abs and IL-2 in the presence of 30 mM malate. Clearly, malate significantly promoted ICOS^+^ eTreg differentiation and,of note, significantly rescued the defect of KO eTreg differentiation (Figure 6, I and J). The addition of malate also consistently rescued the reduced mitochondrial mass by MitoTracker staining and the suppressive function of KO Tregs (Figure 6, K and L, and Supplemental Figure 25). Thus, our results indicate that Zfp335 was required for fatty acid metabolism–mediated eTreg differentiation.

### Zfp335-dependent FAO drives eTreg differentiation.

Tregs exhibit an increase in fatty acid uptake, and FAO-driven OXPHOS is required for Treg-suppressive functions (72). Since Hadha, as a FAO enzyme, was directly targeted by Zfp335 and was also involved in eTreg differentiation, we speculated that reduced ICOS^+^ eTreg differentiation in Zfp335-deficient mice might be due to impaired FAO and a metabolism disorder.

**Figure 7:**
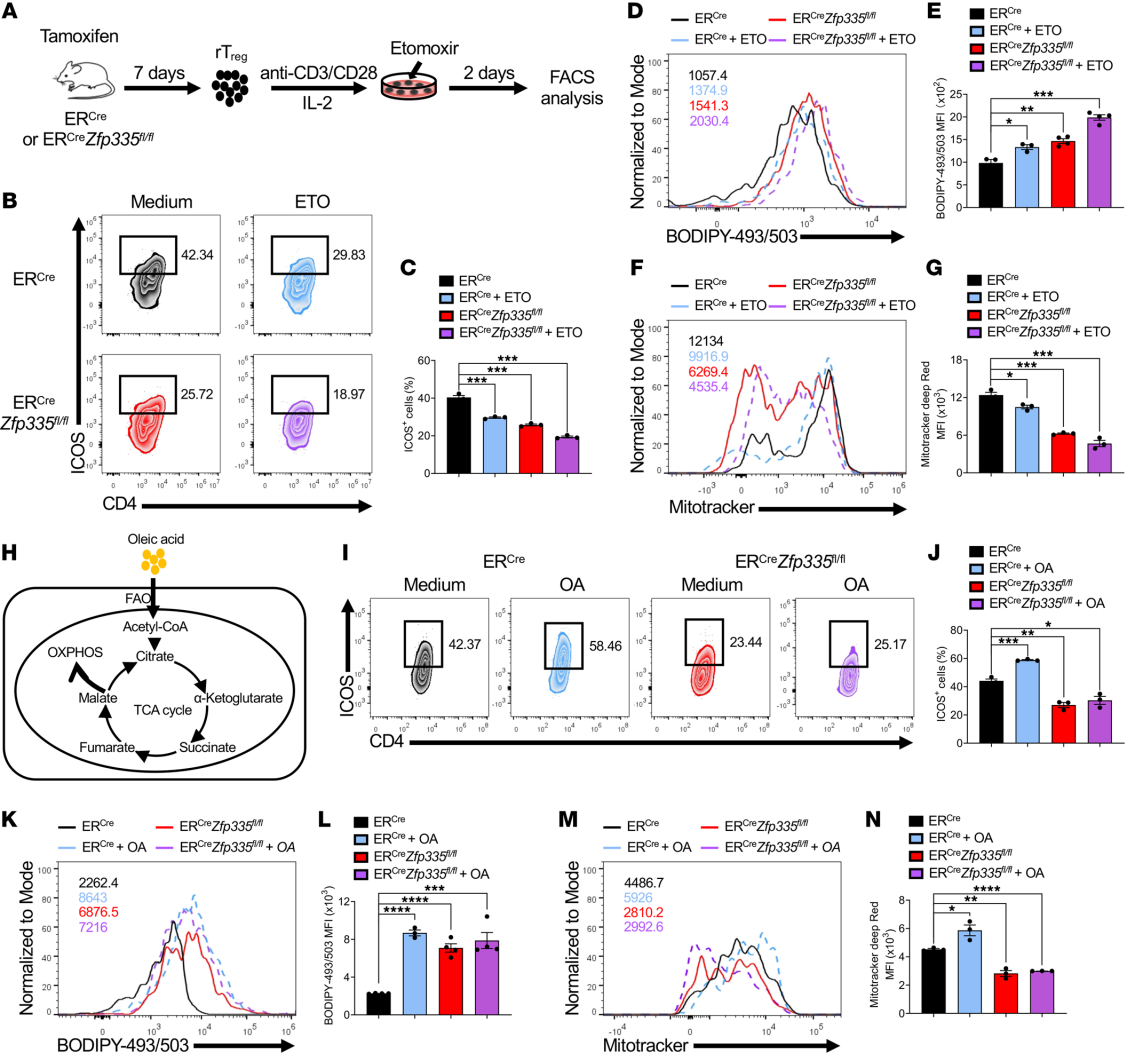
Zfp335-mediated generation of eTregs depends on FAO. (**A**) Schematic diagram of in vitro ETO treatment assay. CD4^+^CD25^+^CD44^–^ICOS^–^ rTregs sorted from ER*^Cre^* and ER*^Cre^* *Zfp335^fl/fl^* mice were activated by anti-CD3/anti-CD28 Abs and IL-2 in the presence or absence of 40 μM ETO. Two days later, the cells were collected and analyzed by FACS. (**B**) Representative FACS plots of ICOS^+^ Tregs in the different groups. (**C**) Frequencies of ICOS^+^ Tregs (*n* = 3). (**D**) Quantification of neutral lipid droplets was evaluated by BODIPY-493/503, and representative FACS plots of BODIPY-493/503 staining are shown. (**E**) MFI of BODIPY-493/503 (*n* = 3–4). (**F**) Representative FACS plots of MitoTracker Deep Red staining. (**G**) MFI of MitoTracker Deep Red (*n* = 3). (**H**) Schematic diagram of the classic pathway of FAO-driven OXPHOS. (**I**–**N**) CD4^+^CD25^+^CD44^–^ICOS^–^ rTregs sorted from ER*^Cre^* and ER*^Cre^* *Zfp335^fl/fl^* mice were activated by anti-CD3/anti-CD28 Abs and IL-2 in the presence or absence of 50 μM oleic acid (OA). Two days later, the cells were collected and analyzed by FACS. (**I**) Representative FACS plots of ICOS^+^ Tregs in the different groups. (**J**) Frequencies of ICOS^+^ Tregs (*n* = 3). (**K**) Quantification of neutral lipid droplets by BODIPY-493/503. (**L**) MFI of BODIPY-493/503 (*n* = 3–4). (**M**) Representative FACS plots of MitoTracker Deep Red staining. (**N**) MFI of MitoTracker Deep Red (*n* = 3). Data represent 2 independent experiments and are shown as the mean ± SEM. *P ≤ 0.05, **P ≤ 0.01, ***P ≤ 0.001, and ****P ≤ 0.0001, by 1-way ANOVA with Tukey’s multiple-comparison test.

To test this, we analyzed the metabolic pathway of fatty acids between WT and KO Tregs. Fatty acid metabolism singscores were higher in cTregs and eTregs from mice of both genotypes compared with rTregs and dysTregs, whereas both cTregs and eTregs from KO mice had reduced fatty acid metabolism singscores compared with the WT group (Supplemental Figure 26). Furthermore, we treated rTregs with 40 μM etomoxir (ETO), an inhibitor of Cpt1, which is the rate-limiting enzyme for FAO (71), under anti-CD3/anti-CD28 Ab culture conditions and IL-2 stimulation for 2 days (Figure 7A). We found that ETO treatment indeed significantly inhibited ICOS^+^ eTreg differentiation in WT (ER*^Cre^*) Tregs (Figure 7, B and C). Of note, KO (ER*^Cre^* *Zfp335^fl/fl^*) rTregs under ETO treatment had a further decreased percentage of ICOS^+^ eTregs (Figure 7, B and C). To further confirm that Zfp335 promotes FAO in Tregs, we examined neutral lipid content via BODIPY-493/503 staining. Consistent with a previous report that inhibition of FAO by ETO could promote lipid droplet accumulation either in Foxp3^+^ or Foxp3^–^ CD4^+^ T cells (73), we observed that both WT and KO Tregs had higher levels of BODIPY-493/503 staining upon ETO treatment (Figure 7, D and E). However, greater lipid droplet accumulation was detected in Zfp335-deficient Tregs compared with Zfp335-sufficient Tregs with and without ETO treatment (Figure 7, D and E). Accordingly, Zfp335-deficient ICOS^+^ eTregs had significantly reduced fatty acid uptake (Supplemental Figure 27). At the same time, we observed a reduction in mitochondrial mass in KO Tregs compared with WT counterparts, and ETO treatment reduced mitochondrial mass in both WT and KO Tregs (Figure 7, F and G). It has been reported that a high concentration of ETO (200 μM) could induce Cpt1-independent off-target effects along with increased fatty acid uptake, whereas a low dose of ETO did not cause an off-target effect (74). In our study, a low dose of ETO caused a reduction in fatty acid uptake in Tregs (Supplemental Figure 28), indicating a lack of off-target effects. Therefore, these data indicate that FAO was defective upon Zfp335 deletion, which in turn caused a decrease in ICOS^+^ eTreg differentiation.

Oleic acid, a long-chain monounsaturated fatty acid, facilitates FAO-driven OXPHOS and supports Treg-suppressive function (75). To gain further insight into whether long-chain fatty acids affect eTreg differentiation driven by Zfp335, we cultured WT and KO rTregs with anti-CD3/anti-CD28 Abs and IL-2, with or without 50 μM oleic acid for 2 days (Figure 7H). Oleic acid indeed increased ICOS^+^ eTreg differentiation (Figure 7, I and J), with accumulation of lipid droplets (Figure 7, K and L) and an increase in mitochondrial mass in WT Tregs (Figure 7, M and N). However, with or without oleic acid supplementation, KO Tregs showed a reduction in ICOS^+^ eTreg differentiation (Figure 7, I and J), with an increase in lipid droplet accumulation (Figure 7, K and L) and a decrease in mitochondrial mass (Figure 7, M and N). Thus, the data indicate that Zfp335 expression was required for oleic acid FAO–mediated eTreg differentiation.

### ZNF335 correlates with human eTreg generation.

**Figure 8:**
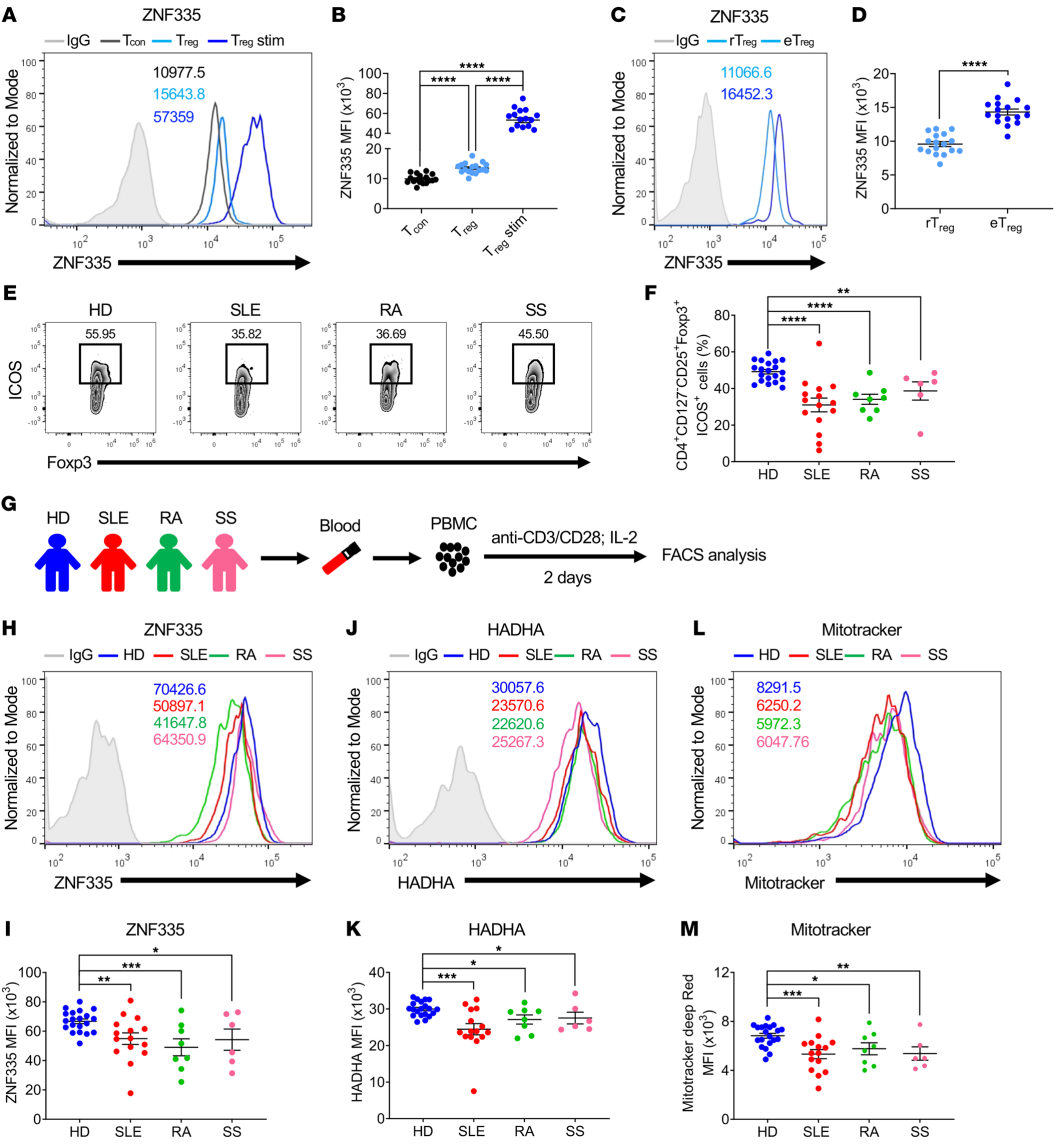
Human ZNF335 expression in eTregs from HDs and patients with autoimmune diseases. (**A** and **B**) ZNF335 expression in Tcons, Tregs, and Tregs from HD PBMCs (*n* = 17) stimulated with anti-CD3/anti-CD28 Abs and IL-2 for 2 days. Representative FACS plots (**A**) and MFI (**B**). (**C** and **D**) ZNF335 expression in rTreg and eTregs from PBMCs of HDs. Representative FACS plots (**C**) and MFI (**D**). (**E**) Representative FACS plots of CD4^+^CD127^–^CD25^+^Foxp3^+^ICOS^+^ cells from HDs (*n* = 20), patients with SLE (*n* = 15), patients with RA (*n* = 8), and patients with SS (*n* = 6). (**F**) Frequencies of CD4^+^CD127^–^CD25^+^Foxp3^+^ICOS^+^ cells in HDs and patients with SLE, RA, or SS. (**G**) PBMCs from peripheral blood of HDs and patients with SLE, RA, or SS were stimulated with anti-CD3/anti-CD28 Abs and IL-2. Two days later, ZNF335 and HADHA expression and MitoTracker Deep Red staining were examined. (**H** and **I**) Representative FACS plots of ZNF335 expression (**H**) and MFI of ZNF335 (**I**) in Tregs from HDs and patients with SLE, RA, or SS. (**J** and **K**) Representative FACS plots (**J**) and MFI of HADHA expression (**K**) in Tregs from HDs and patients with SLE, RA, or SS. (**L** and **M**) Representative FACS plots (**L**) and MFI of MitoTracker Deep Red staining (**M**) in Tregs from HDs and patients with SLE, RA, or SS. Data are shown as the mean ± SEM. *P ≤ 0.05, **P ≤ 0.01, ***P ≤ 0.001, and ****P ≤ 0.0001, by 2-sided, unpaired *t* test (**D**) and Kruskal-Wallis test with the 2-stage step-up procedure of the Benjamini, Krieger, and Yekutieli multiple-comparison test (**B**, **F**, **I**, **K**, and **M**).

To evaluate whether ZNF335 (human ortholog of mouse Zfp335) also plays roles in human Treg differentiation, we first evaluated ZNF335 expression among CD4^+^ T cell populations from healthy PBMCs. In agreement with our findings in mice, Tregs had higher expression of ZNF335 than did Tcons, and ZNF335 levels were dramatically increased in Tregs after stimulation with anti-CD3/anti-CD28 Abs and IL-2 (Figure 8, A and B). Similarly, freshly isolated eTregs (CD4^+^CD25^h​i​^CD45RA^–^) expressed much higher levels of ZNF335 than did rTregs (CD4^+^CD25^+^CD45RA^+^) (Figure 8, C and D), suggesting a critical role of ZNF335 in human eTreg differentiation and function. Notably, the frequency of ICOS^+^ Tregs was gradually increased along with ZNF335 expression (Supplemental Figure 29).

Treg dysfunction, including decreased Treg numbers, particularly in the eTreg subset, and impaired immunosuppressive function are highly associated with autoimmune pathologies (76, 77). We found that patients with systemic lupus erythematosus (SLE), rheumatoid arthritis (RA), or Sjögren’s syndrome (SS) had significantly lower percentages of ICOS^+^ Tregs in the peripheral blood than did healthy donors (HDs) (Figure 8, E and F). We thus examined the relationship between ZNF335 expression and the eTreg population in patients with autoimmune diseases (Figure 8G). Notably, we observed decreased ZNF335 expression in TCR- and IL-2–stimulated Tregs from patients with SLE, RA, or SS (Figure 8, H and I). Importantly, we observed that HADHA, which is the target of Zfp335 and was downregulated in Zfp335-deficient Tregs in mice (Figure 6, A and B), was also downregulated in eTregs from the aforementioned patients (Figure 8, J and K). Consistently, mitochondrial mass was also decreased in patients’ Tregs upon TCR and IL-2 stimulation compared with Tregs from HDs (Figure 8, L and M). Nevertheless, the above results for eTregs, including expression levels of ZNF335 and HADHA and mitochondrial mass, were not observed in CD4^+^ Tcons (Supplemental Figure 30). Thus, the positive correlation between ZNF335 expression and human eTregs suggests a potential role of ZNF335 in human eTreg differentiation, and the reduction in ZNF335, HADHA, and MitoTracker expression in human Tregs may be associated with the pathogenesis of autoimmune diseases.

## Discussion

In this study, we have identified Zfp335 in Tregs as an indispensable factor in maintaining neonatal immune tolerance. Treg-specific deficiency in Zfp335 led to lethal systemic inflammation in mice with severe immunopathology, autoantibodies, effector T cell expansion, overproduction of proinflammatory cytokines, and loss of the Treg signature. Mechanistically, Zfp335 promoted FAO-driven metabolism by direct regulation of the expression of the FAO enzyme gene *Hadha*. Importantly, we demonstrate the positive correlation between *ZNF335/HADHA* expression, metabolic activity, and the human eTreg population.

Defects in the proportion, stability, and function of Tregs break immune tolerance and cause autoimmune syndromes (1, 10). In our study, Zfp335 deficiency impaired the immunosuppressive function of Tregs both in vitro and in vivo. This conclusion is supported by the evidence of the failure of Tregs in suppressing effector T cell expansion and proinflammatory cytokine production. In addition, the fact that the transfer of WT Tregs into 2-day-old KO pups sufficiently prevented autoimmune symptoms in KO mice further supports our conclusion. However, the frequencies and numbers of Tregs in the thymus and peripheral lymph tissues were comparable between *Foxp3^Cre^* and *Foxp3^Cre^* *Zfp335^fl/fl^* mice at 1 week of age. Thus, the lethal inflammation that developed in *Foxp3^Cre^* *Zfp335^fl/fl^* mice was initiated by the quality rather than the quantity of Treg defects.

Our transcriptomic analysis uncovered evidence that signaling pathways involved in Treg differentiation were largely downregulated, while those associated with lymphocyte activation and inflammatory responses were upregulated in Zfp335-deficient Tregs. We also detected high expression of Th1 and Th2 signature cytokines and transcription factors in Zfp335-deficient Tregs. These changes demonstrate an apparent instability of Tregs in the *Foxp3^Cre^* *Zfp335^fl/fl^* mice. However, Zfp335 deficiency failed to significantly affect Foxp3 protein expression in Tregs, excluding the possibility of direct regulation of Foxp3 by Zfp335. Therefore, the instability and defective function of Tregs in Zfp335-deficient mice are most likely due to an alternative effect.

Indeed, our comprehensive scRNA-Seq of Tregs from asymptomatic KO mice identified 4 Treg clusters including a distinct dysTreg population annotated by minimum expression of prominent genes critical for Treg differentiation and suppressive function, with enriched expression of inflammation-related genes. Strikingly, examination of Treg distribution revealed an unexpected loss of eTregs but an accumulation of dysTregs in *Foxp3^Cre^* *Zfp335^fl/fl^* mice. The trajectory analysis of the 4 Treg clusters further showed that rTregs could differentiate into cTregs, eTregs, and dysTregs. Given the dramatic imbalance between dysTreg and eTreg populations in *Foxp3^Cre^* *Zfp335^fl/fl^* mice, we believe that Zfp335 controls eTreg lineage differentiation. Loss of Zfp335 dampened the generation of eTregs in neonatal mice, which caused lethal inflammation. These findings emphasize the importance of eTreg regulation in establishing tolerance under healthy conditions as well as in preventing autoreactive responses in an inflammatory environment. Therefore, eTreg-based immunotherapy should be considered for autoimmune diseases.

Cellular metabolism is extensively involved in Treg differentiation and suppressive function (63, 78, 79). Recent studies reported that FAO is required for the suppressive function of Treg and the prevention of autoimmunity (80, 81), while oleic acid could increase Foxp3 expression and Treg function in patients with multiple sclerosis (75). However, it is unclear whether FAO affects the components of the Treg population, especially the differentiation of eTregs. Here, we found that utilization of oleic acid as metabolic fuel specifically promoted eTreg differentiation, which was dependent on Zfp335 expression. Consistently, inhibition of FAO by ETO decreased the generation of eTregs. Of note, it has been shown that a high concentration of ETO (200 μM) leads to Cpt1-independent off-target effects in tumor cells by inhibiting complex I of the electron transport chain (74). The off-target effects of a high concentration of ETO exhibited the increase in fatty acid uptake, however, a low dose of ETO (40 μM) caused a reduction in fatty acid uptake in Tregs in our study, indicting a lack of off-target effects. Thus, FAO was required for eTreg differentiation in a Zfp335-dependent manner.

By comparing the biological pathways between eTregs and dysTregs, we found that PI3K/Akt/mTOR and OXPHOS signaling pathways were markedly reduced in dysTregs. Unlike in effector T cells, which rely on glycolysis, OXPHOS is the main metabolic pathway in Tregs for energy production and immunosuppressive function (34, 82). In addition, activation of the PI3K/Akt/mTOR pathway promotes Treg amplification, as inhibition of the PI3K/Akt pathway selectively suppresses Treg proliferation (83). Here, we identified a number of genes involved in OXPHOS and PI3K/Akt/mTOR signaling that are downregulated in Zfp335-deficient eTregs. We performed ChIP-Seq analysis, which further confirmed a subset of genes involved in metabolism as direct targets of Zfp335. In particular, *Hadha*, a key FAO enzyme involved in the OXPHOS signaling pathway (84), was shown to be directly targeted by Zfp335 and involved in regulating eTreg differentiation. Following FAO, acetyl-CoA enters into the mitochondrial TCA cycle to fuel mitochondrial OXPHOS. Our results showed that the supplementation of malate, one of the TCA intermediates, could restore impaired eTreg differentiation in Zfp335-deficient mice, further confirming that Zfp335 controls eTreg differentiation through FAO-driven OXPHOS.

Zfp335, serving as a transcription factor, was initially found to play important regulatory roles in early embryonic development and neurogenesis (43). Recently, it has also been shown that Zfp335 is involved in regulating multiple aspects of T cell biology. Zfp335 is not only required for the maturation and migration of thymocytes by direct regulation of Ankle2 expression (46), but also controls DN to DP transition through the regulation of TCR-β expression as well as thymocyte survival by targeting Bcl6/Rorc (47). More recently, we reveal that Zfp335 is also involved in memory CD8^+^ T cell differentiation by direct regulation of TCF7 expression (47). The findings demonstrated here that Zfp335 promoted FAO metabolism–mediated eTreg differentiation by direct regulation of gene expression of the FAO enzyme *Hadha*. Although Zfp335 is also a component of the trithorax H3K4–methylation complex, we did not see obvious H3K4 trimethylation at the promoter loci of the above-mentioned target genes. Therefore, Zfp335 is an essential transcriptional factor and involved in regulating different cell lineages and the developmental stages of T cells through distinct mechanisms.

In summary, we have unveiled a previously unrecognized role of Zfp335 in establishing neonatal immune tolerance and preventing lethal autoimmunity. Mechanistically, Zfp335 controlled eTreg lineage commitment through the regulation of FAO by targeting Hadha. Our findings provide new insights into the understanding of the heterogeneity, suppressive function, and underlying regulatory mechanisms of Tregs. Our results identify Zfp335 as a potential therapeutic target for the manipulation of eTreg function in human autoimmune diseases.

## Methods

### Mice.

Eight-week-old *Zfp335^fl/fl^*, *Foxp3^Cre^*, ER*^Cre^*, and *CD45.1*^+^ mice were purchased from The Jackson Laboratory. Eight-week-old *Rag2^–/–^* mice were purchased from the Beijing Huafukang Biotechnology Company. *Foxp3^Cre^* mice contain an internal ribosomal entry site (IRES) and a YFP fused to a codon-optimized Cre Recombinase (iCre) sequence downstream of the internal stop codon of the *Foxp3* gene. *Foxp3^Cre^* mice were crossed with *Zfp335^fl/fl^* and *Zfp335^+/+^* mice to generate *Foxp3^Cre^* (WT) mice, heterozygous *Foxp3*^Cre/+^ *Zfp335^fl/fl^* mice, and *Foxp3^Cre^* *Zfp335^fl/fl^* (KO) mice. ER*^Cre^* mice express a fusion protein containing an estrogen receptor (ER) ligand-binding region mutant (ERT) and Cre recombinase. Cre was inactive in cytoplasm without tamoxifen induction. When induced by tamoxifen, the tamoxifen metabolite 4-OHT (estrogen analog) binds to ERT, which enables Cre-ERT to enucleate and exert Cre recombinase activity. ER*^Cre^* mice were crossed with *Zfp335^fl/fl^* mice and *Zfp335^+/+^* mice to generate ER*^Cre^* *Zfp335^fl/fl^* (KO) mice and ER*^Cre^* (WT) mice. Tamoxifen (10 mg/mL) dissolved in corn oil was administered to 6-week-old mice on days 1, 3, and 5 (100 μL/injection), and the mice were sacrificed on day 7. All mice were housed under specific pathogen–free conditions at the Xi’an Jiaotong University Division of Laboratory Animal Research.

### Human samples.

All patients and HDs enrolled in this study were recruited from the department of Rheumatology and Immunology at the First Affiliated Hospital of Xi’an Jiaotong University. A total of 15 patients with SLE (*n* = 1 male, *n* = 14 females; mean age, 32.3 ± 15 years); 8 patients with RA (*n* = 1 male, *n* = 7 females; mean age, 46.3 ± 21.1 years); 6 patients with SS (*n* = 6 females; mean age, 47.2 ± 12.8 years); and 20 HDs were enrolled in the study (*n* = 7 males, *n* = 13 females; mean age, 44.4 ± 10.5 years). A diagnosis of SLE was based on the 2012 Systemic Lupus International Collaborating Clinics criteria (SLICC-12) (85). A diagnosis of RA was based on the 2010 American College of Rheumatology/European League against Rheumatism criteria (86). A diagnosis of SS was based on the 2016 American College of Rheumatology/European League against Rheumatism criteria (87).

### T cell transfer–based colitis.

CD45.1^+^CD4^+^CD45RB^hi^ naive T cells from CD45.1^+^ WT mice, and CD45.2^+^CD4^+^CD25^+^ Tregs from ER*^Cre^* mice or ER*^Cre^* *Zfp335^fl/fl^* mice treated with 3 doses of tamoxifen were sorted on a FACSAria II(BD Bioscience) cell sorter. Naive T cells (5 × 10^5^) mixed with or without 2.5 × 10^5^ Tregs were i.v. injected into *Rag2^−/−^* recipient mice. Mouse weights were monitored daily for 24 days, and tissues were collected at the endpoint of the experiment.

### Seahorse analysis.

The cellular OCR was measured using the Seahorse XFe Analyzer (Agilent Technologies). CD4^+^CD25^+^CD44^–^ICOS^–^ rTregs purified from ER*^Cre^* and ER*^Cre^* *Zfp335^fl/fl^* mice were sorted and activated with 5 μg/mL anti-CD3, 2 μg/mL anti-CD28, and 500 U/mL IL-2. After a 12-hour stimulation, 4 × 10^5^ activated Tregs were harvested, resuspended in Seahorse XF DMEM (supplemented with 10 mM glucose, 1 mM pyruvate, and 2 mM glutamine), and seeded in Cell-Tak (Corning) precoated Seahorse 96-well plates. The OCR was assessed under basal conditions and in response to 1.5 μM oligomycin, 2 μM fluorocarbonyl cyanide phenylhydrazone (FCCP), 1 μM rotenone, and antimycin A.

### scRNA-Seq data analysis.

The gene expression matrices of all samples were imported into Seurat (version 4.0.0), version 4 (88), and merged for subsequent analyses. Filtering steps were carried out to exclude low-quality cells. The DoubletFinder package (version 2.0.3) was used to detect double cells. The ratio of double cells in the KO and WT groups was 5% and 8%, respectively. Cells with fewer than 300 genes and identified as “doublet” were discarded, and cells with a high fraction of mitochondrial genes (>5%) were removed. As a result, a total of 19,215 cells (WT = 7,124; KO = 12,091) with a median of 1,358 genes were included in the analyses. After scaling data with ScaleData, a PCA dimensionality reduction (RunPCA) was performed, and the first 30 PCs were selected to construct a shared nearest neighbor (SNN) graph using FindNeighbors. To visualize the clustering results, a nonlinear dimensional reduction was performed with the UMAP method, and cluster biomarkers were found by the FindAllMarkers function in the Seurat package.

Additional methodological information is provided in the Supplemental Methods.

### Statistics.

Statistical analysis was performed using GraphPad Prism 7 (GraphPad Software). Significant differences were assessed by 2-tailed Student’s *t* test, 1-way ANOVA, or Kruskal-Wallis test. Data are presented as the mean ± SEM. All bar graphs include means with error bars to show the distribution of the data. A *P* value of 0.05 or less was considered statistically significant.

### Study approval.

All animal procedures were approved by the IACUC of Xi’an Jiaotong University (approval no. 2017-1012). The studies using human samples were approved by the ethics committee of the First Affiliated Hospital of Xi’an Jiaotong University (Xi’an, China), and all research methods were conducted in compliance with the Declaration of Helsinki of 1964 and relevant regulations and guidelines. Written informed consent was obtained from all study participants.

### Data availability.

The scRNA and RNA-Seq data generated in this study are deposited in the NCBI’s Gene Expression Omnibus (GEO) database (GEO GSE189058, GSE189041, and GSE189077). The ChIP-Seq data for this study are deposited in the GEO database (GEO GSE189076). Values for all data points shown in graphs are reported in the Supplemental Supporting Data Values file.

## Author contributions

BZconceptualized the study and designed experiments. XW, LS, BY, and WL carried out most of the experimental work. CZ analyzed scRNA-Seq data under the supervision of BZ. LZ, Y Sun, XS, XY, Yafeng Gao, JS, Y Su, AJ, HL, LL, Yang Gao, and XJ helped with mouse experiments. BJ and DL provided the human samples. LH contributed to scientific discussions. WJC provided crucial scientific input and edited the manuscript. BZ, XW, LS, and CZ wrote the manuscript. The order of the co–first authors was assigned on the basis of their efforts and contributions to the study.

## Supplementary Material

Supplemental data

Supplemental table 1

Supplemental table 2

Supplemental table 3

Supplemental table 4

Supplemental table 5

Supporting data values

## Version 1 10/16/2023

Electronic publication
