# Supplementary material for: Zfp335 establishes eTreg lineage and neonatal immune tolerance by targeting Hadha-mediated fatty acid oxidation
Source: J Clin Invest. 2023 Oct 16;133(20):e166628. doi: 10.1172/JCI166628 (PMC10575732; doi:10.1172/JCI166628)
Supplement: Supplemental data [file jci-133-166628-s157.pdf]

Materials and methods

Antibodies

Single-cell preparations were stained with the monoclonal antibodies purchased from Biolegend (San Diego, CA, USA): APC/Cy7 anti-mCD4 (clone GK1.5), PE/Cy5 anti-mCD4 (clone GK1.5), PB anti-mCD8α (clone 53-6.7), PE/Cy7 anti-mCD8α (clone 53-6.7), PE/Cy5 anti-mCD8 (clone GK1.5),PE/Cy7 anti-mCD44 (clone IM7), APC anti-mCD62L (clone MEL-14), PE/Cy7 anti-mIFN-γ (clone XMG1.2), PE anti-mIL-17 (clone TC11-18H10.1), PB anti-mIL-17 (clone TC11-18H10.1), PB anti-mIL4 (clone 11B11), PE anti-mCD25 (clone PC61), PE/Cy5 anti-mCD25 (clone PC61), APC anti-mFoxp3 (clone MF-14), PB anti-mFoxp3 (clone MF-14), APC anti-mCD45RB (clone RA3-6B2), PE/Cy7 anti-mLy6c (clone RB6-8C5), PE anti-mCD11b (clone M1/70), PE/Cy7 anti-mTbet (clone 4B10), PE/Cy5 anti-mGATA-3 (clone 16E10A23), APC/Cy7 anti-mCD45.1 (clone A20), PE anti-mCD45.2 (clone 104), APC anti-mCD45.2 (clone 104), PE/Cy5 anti-mCD3 (clone 17A2), PE/Cy5 anti-mCD45R/B220 (clone RA3-6B2), PE/Cy5 anti-mCD11b (clone M1/70), PE/Cy5 anti-mCD11c (clone N418), PE/Cy5 anti-mCD11b (clone M1/70), APC/Cy7 anti-mCD19 (clone 6D5), APC anti-mGL7 (clone), PE anti-mFas (clone SA367H8), PE anti-mPD1 (clone 29F.1A12), APC anti-mCXCR5 (clone L138D7), PE anti-mICOS (clone 15F9), PE/Cy5 anti-mICOS (clone 15F9), PE/Cy7 anti-mCXCR3 (clone S18001A), APC/Cy7 anti-hCD4 (clone RPA-T4), PE/Cy7 anti-hCD25 (clone M-

A251), PE anti-hFoxp3 (clone 259D), FITC anti-hCD127 (clone A019D5), PE/Cy5 anti-hCD45RA (clone HI100), APC anti-hCD45RA (clone HI100), PB anti-hICOS (clone C398.4A), Pacific Blue™ anti-Annexin V (Cat # 640918), and the Fixation/Permeabilization Solution Kit (Cat # 554722). PE anti-Ki67 monoclonal antibody (clone SolA15) and Transcription Factor Fixation/Permeabilization Concentrate and Diluent were purchased from eBioscience (San Diego, CA, USA).

Flow cytometry

For analysis of surface markers, cells were stained in PBS containing 2% FBS on ice for 30min. For analysis of intracellular cytokine staining, cells were stimulated for 4 h *in vitro* with PMA/Ionomycin in the presence of brefeldin A and monensin. The stimulated cells were fixed and permeabilized using a Fixation/Permeabilization Solution Kit (Biolegend).

For analysis of mitochondrial mass, membrane potential and ROS production, cells were stained with 100nM MitoTracker deep Red, 500nM Tetramethylrhodamin-Ethylester (TMRE) and 2.5 μM MitoSox for 30 min at 37℃°, respectively (Invitrogen). Lipid uptake and neutral lipid content were measured using the green fluorescent fatty acid BODIPY-FL-C16 (Invitrogen) and BODIPY-493/503 reagent (Shanghai maokang biotechnology Co., LTD).

T_{reg} cells were incubated with 3uM BODIPY-FL-C16 and 2 μM BODIPY-493/503 for 30 min at 37℃°. After the incubation, the cells were washed in RPMI 1640 medium with 2% FBS and continued for staining surface markers at room temperature in dark.

All samples were analyzed using a CytoFLEX flow cytometer (BECKMAN COULTER). FlowJo software and CytExpert software were used for data analysis. Cells were sorted with a FACSAria (BD Biosciences).

RNA isolation and quantitative PCR (qPCR)

Total RNA was extracted with a RNeasy Mini Kit (Qiagen), according to the manufacturer's instruction. cDNA was reverse transcribed using the cDNA synthesis kit (TOYOBO) and amplified with SYBR Green RT-qPCR Mastermix (GenStar) at StepOnePlusTM Real-Time PCR System (ThermoFisher). Primer sequences used in this study were summarized in Supplemental table 5.

HE staining and histopathology

Lungs, livers, kidneys, ears, pancreas, salivary glands, thymi, hearts and lacrimal glands were removed from 3-week-old WT and KO mice. Colons were removed from 3-week-old WT and KO mice or mice as mentioned in colitis model. Samples were formalin fixed, paraffin embedded and stained with

haematoxylin and eosin before tissue histology. Photomicrographs were taken at x20 or x5 magnifications.

ELISA for autoantibody

Serum samples were collected from 3-week-old WT and KO mice. Autoantibodies (anti-dsDNA) were measured using a detection kit from Alpha Diagnostic International (5110) according to the manufacturer's instructions.

T_{reg} suppression assay in vitro

$CD4^+$ T cells were enriched from spleen and LN of WT mice using MojoSort™ Mouse CD4 Naïve T Cell Isolation Kit (Biolegend). $CD4^+CD25^+YFP^+$ T_{reg} cells and $CD4^+CD25^-CD44^-CD62L^+$ naïve T cells were sorted on FACSAria Ⅱ (BD Bioscience) cell sorter. Naïve T cells were labelled with 5μM CellTraceViolet (CTV) (Biolegend) at 37°C for 15min, followed by three washes, and mixed with WT or KO $CD4^+CD25^+YFP^+$ T_{reg} cells in a 96-well plate stimulated with purified 2μg/ml anti-CD3 antibody in RPMI medium supplemented with 10% FBS (HyClone), 1% penicillin and streptomycin and 50 μM β-mercaptoethanol (Sigma-Aldrich). After 60h, the proliferation of conventional T cells was analyzed by CytoFLEX flow cytometer (BECKMAN COULTER).

T_{reg} cell adoptive transfer assay

T_{reg} cells were sorted using FACSAria Ⅱ (BD Bioscience) cell sorter. For T_{reg} cell functionality, $1.8x10^6$ WT T_{reg} cells from $CD45.1^+$ mice were sorted and intraperitoneal (i.p.) injected into 2-day-old KO (*Foxp3*^{Cre}*Zfp335*$^{fl/fl}$) pups for 19 days.

Retroviral transduction of T_{reg} cells

Retroviruses were produced from 293-derived BOSC cells transfected with tdTomato control (Mock), tdTomato-*Ndufa4*, tdTomato-*Hadha* and tdTomato-*Actr2* plasmids. For retroviral transduction, $CD4^+YFP^+CD44^-ICOS^-$ T_{reg} cells were sorted and activated with Dynabeads Mouse T-Activator CD3/CD28 (ThermoFisher) at a bead-to-cell ratio of 2:1 and 500U/ml IL-2. Transduction was performed 20 hours after activation by centrifugation (2500rpm for 1.5 hours at 37℃) in the presence of retroviral supernatants, 8μg/ml polybrene and 500U/ml IL-2. After spin infection, supernatants were replaced by RPMI medium with 10% FBS supplemented with 500U/ml IL-2. T_{reg} cells were collected 4 days after transfection for ICOS expression by flow cytometry.

Malate supplementation in vitro

For malate treatment in vitro, resting T_{reg} (rT_{reg}) cells purified from Tamoxifen treated-ER^{Cre} and ER^{Cre}*Zfp335*$^{fl/fl}$ mice were sorted and activated with 5μg/ml anti-CD3 Ab, 2μg/ml anti-CD28 Ab and 500U/ml IL-2, and supplemented with

or without 30 mM malate. 72h later, cells were collected and analyzed by flow cytometry.

In vitro Etomoxir treatment

To measure the effect of FAO inhibitor etomoxir (ETO; Selleck) on effector T_{reg} (eT_{reg}) differentiation, sorted resting T_{reg} (rT_{reg}) cells were activated by 5μg/ml anti-CD3 Ab, 2μg/ml anti-CD28Ab and 500U/ml IL-2 for 2 days in the presence or absence of 40uM ETO.

In vitro fatty acid supplementation

Sodium oleate (Sigma) was dissolved in PBS and stocked at 25 mM. Oleate was then dissolved by heating in a metal bath at 70C° and conjugated with RPMI 1640 medium supplemented with 1.6% FA-free BSA. Purified rT_{reg} cells were activated by 5μg/ml anti-CD3 Ab, 2μg/ml anti-CD28 Ab and 500U/ml IL-2 for 2-3 days in the presence or absence of 50uM sodium oleate. The cells were then collected and applied for flow cytometry analysis.

Chromatin Immunoprecipitation

2.5×10^7 T_{reg} cells were sorted from the lymph nodes of WT mice on FACSAria II (BD Bioscience) cell sorter. Millipore 17–10085 Chromatin Immunoprecipitation (ChIP) kit and anti-Zfp335 antibody (Novus) were used in the ChIP assay. Immunoprecipitated DNA was used for Illumina ChIP-seq

sample preparation. In brief, 2.5×10^7 cells were crosslinked to chromatin with 1% formaldehyde. Reaction was stopped with 0.125M glycine. The cells were resuspended in cold nuclear lysis buffer and the chromatin was sonicated to yield fragments of ~300-500bp size, followed by overnight incubation with immunoprecipitation–grade anti-Zfp335 antibody and Magnetic Protein A/G Beads. The following day, beads were sequentially washed by low-salt, high-salt, LiCl, and TE buffers. Bound complexes were eluted in 150μl of elution buffer at 62°C for 2h with shaking, followed by reversal of formaldehyde crosslinking at 95°C for 10 minutes. DNA was eventually purified with spin columns.

Immunoprecipitated DNA concentration was detected by the Qubit DNA broad range assay in the Qubit Fluorometer (Invitrogen). 10ng immunoprecipitated DNA was prepared for sequencing using the Illumina ChIP-seq sample preparation protocol. The library products were enriched quantified and finally sequenced on Novaseq 6000 sequencer (Illumina) with PE150 model. Raw sequencing data were filtered by Trimmomatic (version 0.36). FastQ reads were aligned to the ensemble mouse genome (GRCm38) with STAR software (version 2.5.3a) using default settings. The MACS2 software (Version 2.1.1) was used to process peak calling. The library products corresponding to 200-500 bps were enriched, quantified and finally sequenced on Novaseq 6000

sequencer (Illumina) with PE150 model. Genomic graphs were generated and viewed with the IGV (Integrative Genomics Viewer).

Bulk RNA-seq data analysis

Total RNA was isolated form $CD4^{+}CD25^{+}YFP^{+}$ T_{reg} cells of 2-week-old *Foxp3*^{Cre} and *Foxp3*^{Cre}*Zfp335*$^{fl/fl}$ mice and used for RNA sequencing analysis. Firstly, the bulk RNA-seq data were filtered by using SAOPnuke (version 1.5.6) (1) with parameters "-l 15 -q 0.2 -n 0.05 -Q 2". After removing low-quality bases RNAs, the clean data were mapped to mouse genome (mm10) by using HISAT2 (2) with parameters "-k 1 -p 4 -q --no-unal --dta --un-conc-gz". Then the expression levels of each gene were calculated by the transcripts per kilobase of exon model per million mapped reads (TPM) by using StringTie (3) with parameters "-t -C -e -B -A -p 1". The final TPM matrix of all samples was used for subsequent analysis. A 1.5-fold variance in expression levels, a P value less than 0.05, and an adjusted P value less than 0.1 were used as cutoffs to define differentially expressed genes. The P value and adjusted P value were calculated using R software (DESeq2) (4).

Single-cell RNA sequencing processing

The spleens were dissociated into single-cell suspensions with the following procedure: Spleens were processed with the flat end of a syringe in a 100 mm

culture dish containing 5 ml cold FACS buffer (2% FBS in PBS), then passed through a 70 μm cell strainer into a 15 ml tube. Cells were centrifuged to remove the supernatant. Cell pellets were treated with 1ml ACK (Ammonium-Chloride-Potassium) Lysing Buffer to remove the red blood cells. After washing with 10 ml cold FACS buffer, the remaining cells were stained with 7AAD (Part 76332; Lot B226294 Biolegend) for 30 min at 4 °C before flow cytometric sorting using FACS Aria II Cell Sorter (BD Biosciences). The sorted $CD4^{+}YFP^{+}7AAD^{-}$ cells with a viability higher than 90% were used for 10X genomics scRNA-seq. Furthermore, the single-cell library preparation was constructed using 10X Chromium Single Cell V3 Reagent Kits according to the manufacturer's protocol. Cell Ranger (V5.0.1, https://support.10xgenomics.com/) was used to process scRNA-seq data and generate the matrix data containing gene counts for each cell per sample. Briefly, the 10X sequencing data were mapped to the mouse genome (mm10) which downloaded from 10X Genomics and generated the unique molecular identifiers (UMI) matrix of each cell by using Cell Ranger (version 5.0.1) count pipeline.

GO, KEGG and Gene Set Enrichment Analysis

Gene Ontology (GO) and Kyoto Encyclopedia of Genes and Genomes (KEGG) enrichment analyses were performed using clusterProfiler (V3.18) package using genes specifically expressed in indicated T_{reg} cell cluster. Gene Set

Enrichment Analysis (GSEA) analysis was performed for each cell subpopulation using the scaled gene expression matrix and GSEA package (V.4.1) available at Molecular Signatures Database (MSigDB, https://www.gseamsigdb.org/gsea/downloads.jsp) with default parameters.

Single-cell trajectory analysis

To reveal the differentiation relationship of various T_{reg} subsets, Monocle (v3) (5) was used for pseudotime analysis. The Seurat object was converted to a Monocle3 object using as.cell_datA_set function. Then cluster_Cells and learn_graph functions were used to construct developmental trajectories in UMAP. The get_earliest_principal_node helper function as was used to assign a node for which the highest fraction of closest cells belonged to the rT_{reg} cluster as the root node. Then, the 'order_cells' function was used to order cells and the plot_cells function was used to visualize the trajectory in two-dimensional spaces.

Hallmark Gene Set score quantification

To score individual cells for Hallmark pathway activities, we used multi-previous described methods analyzing different T_{reg} subsets data. Firstly, the mouse Hallmark Gene Sets transformed from the human genes were used from msigdbr package, and gene sets were then used to score each cell. To

eliminate the bias of sample background information, we selected gene set enrichment analysis methods based on single cell gene expression ranking AUCell (6), UCell (7), singscore (8) and ssGSEA (9). Of note, ssGSEA cancels the final standardization step, making it closer to the gene set enrichment analysis of a single cell. In addition, to evaluate whether the gene set is enriched in a certain cell subpopulation, we calculated the differential gene set in the enrichment score matrix by Wilcox test (the filter criterion for differential genes is that the P value after correction is less than 0.05). Finally, we used the rank aggregation algorithm (RRA) in the RobustRankAggreg package (10) (version 1.1.0) to comprehensively evaluate the results of the difference analysis, and screen out the genes that are significantly enriched in most gene set enrichment analysis methods Set (the filter criterion for comprehensive evaluation is P value less than 0.05).

References

1. Chen Y, Chen Y, Shi C, Huang Z, Zhang Y, Li S, et al. SOAPnuke: a MapReduce acceleration-supported software for integrated quality control and preprocessing of high-throughput sequencing data. *Gigascience.* 2018;7(1):1-6.
2. Pertea M, Kim D, Pertea GM, Leek JT, and Salzberg SL. Transcript-level expression analysis of RNA-seq experiments with HISAT, StringTie and Ballgown. *Nat Protoc.* 2016;11(9):1650-67.
3. Pertea M, Pertea GM, Antonescu CM, Chang TC, Mendell JT, and Salzberg SL. StringTie enables improved reconstruction of a transcriptome from RNA-seq reads. *Nat Biotechnol.* 2015;33(3):290-5.
4. Love MI, Huber W, and Anders S. Moderated estimation of fold change and dispersion for RNA-seq data with DESeq2. *Genome Biol.* 2014;15(12):550.

5. Cao J, Spielmann M, Qiu X, Huang X, Ibrahim DM, Hill AJ, et al. The single-cell transcriptional landscape of mammalian organogenesis. *Nature.* 2019;566(7745):496-502.
6. Aibar S, Gonzalez-Blas CB, Moerman T, Huynh-Thu VA, Imrichova H, Hulselmans G, et al. SCENIC: single-cell regulatory network inference and clustering. *Nat Methods.* 2017;14(11):1083-6.
7. Andreatta M, and Carmona SJ. UCell: Robust and scalable single-cell gene signature scoring. *Comput Struct Biotechnol J.* 2021;19:3796-8.
8. Foroutan M, Bhuva DD, Lyu R, Horan K, Cursons J, and Davis MJ. Single sample scoring of molecular phenotypes. *BMC Bioinformatics.* 2018;19(1):404.
9. Hanzelmann S, Castelo R, and Guinney J. GSVA: gene set variation analysis for microarray and RNA-seq data. *BMC Bioinformatics.* 2013;14:7.
10. Kolde R, Laur S, Adler P, and Vilo J. Robust rank aggregation for gene list integration and meta-analysis. *Bioinformatics.* 2012;28(4):573-80.

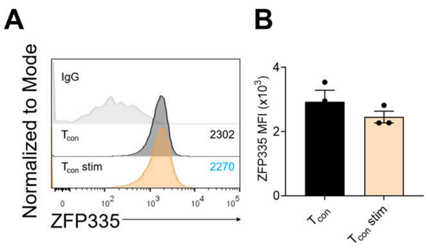

Supplemental Figure 1. ZFP335 expression in CD4$^+$ conventional cells (T_{con}). (**A**) Histograms for ZFP335 expression in T_{con} cells with or without stimulation with anti-CD3/CD28 Abs and IL-2 for 2 days. (**B**) Mean fluorescence intensity (MFI) of ZFP335 for (**A**) (n = 3). Data are representative of three independent experiments shown as the mean ± s.e.m. Statistical analysis is depicted as two-sided, unpaired *t* test.

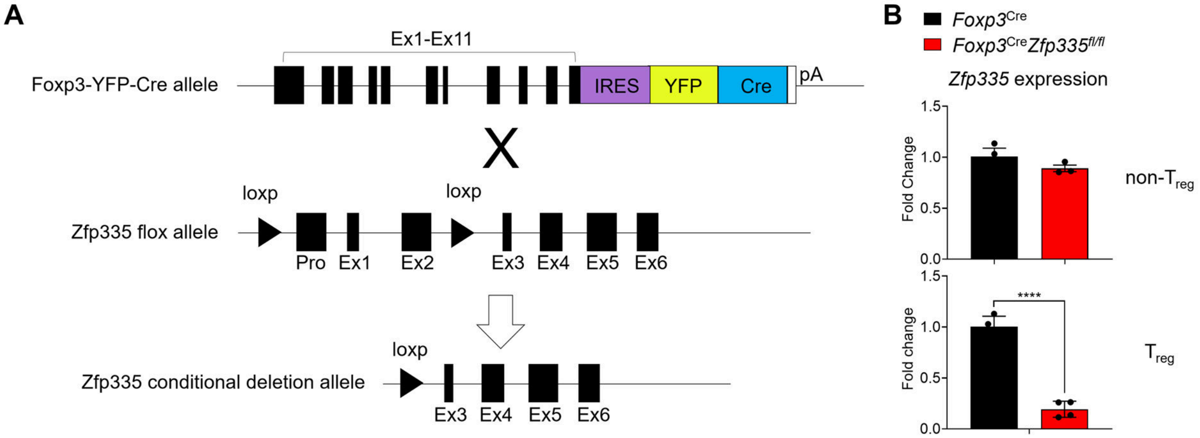

Supplemental Figure 2. Verification of T_{reg}-specific Zfp335-deficient mouse strain. (**A**) The strategy for generating T_{reg}-specific Zfp335-deficient mice. *Zfp335*$^{fl/fl}$ mice were crossed with *Foxp3*$^{YFP\text{-}Cre}$ (*Foxp3*^{Cre}, WT) mice to generate *Foxp3*$^{YFP\text{-}Cre}$*Zfp335*$^{fl/fl}$ (*Foxp3*^{Cre}*Zfp335*$^{fl/fl}$, KO) mice. (**B**) Relative changes of mRNA expression of *Zfp335* in $CD4^+YFP^-$ non-T_{reg} and $CD4^+CD25^+YFP^+$ T_{reg} cells from WT and KO mice (n = 3~4). Data are representative of three independent experiments shown as the mean ± s.e.m. Statistical analysis is depicted as two-sided, unpaired t test; ****$P \leq 0.0001$.

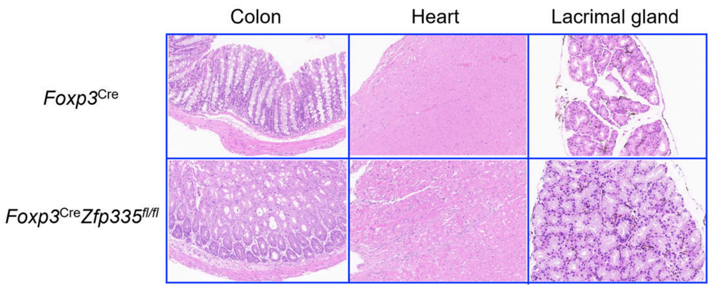

Supplemental Figure 3. Histological analysis of mouse organs. Hematoxylin and eosin (H&E) staining of colon, heart and lacrimal gland sections from 3-week-old WT and KO mice (magnification, x20; Scale bar = 50 μm).

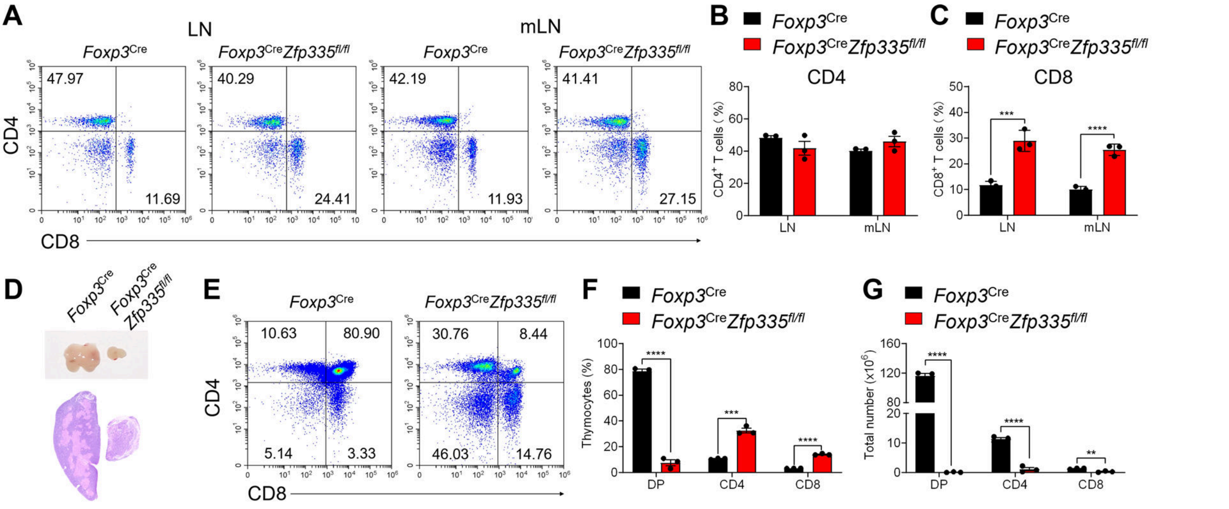

Supplemental Figure 4. Effect of Zfp335 deletion on cell proportions in thymi and peripheral lymphoid organs. (**A**) Representative FACS plots of CD4 and CD8 expression in LN (Left) and mLN (Right) from 3-week-old WT and KO mice. **(B** and **C)** Statistical frequencies of $CD4^+$ (**B**) and $CD8^+$ (**C**) T cells in LN and mLN (*n* = 3). (**D**) Representative image (Top) and HE staining (Bottom) of thymi from 3-week-old WT and KO mice (magnification, x2.5; Scale bar = 500 μm). (**E**) Representative FACS plots of CD4 and CD8 expression in thymocytes from 3-week-old WT and KO mice. (**F** and **G**) Statistical frequencies (**F**) and cell numbers (**G**) of $CD4^+CD8^+$ (DP), $CD4^+$ and $CD8^+$ thymocytes (*n* = 3). Data are representative of three independent experiments shown as the mean ± s.e.m. Statistical analysis is depicted as two-sided, unpaired *t* test; $^{**}P \leq 0.01$, $^{***}P \leq 0.001$, $^{****}P \leq 0.0001$.

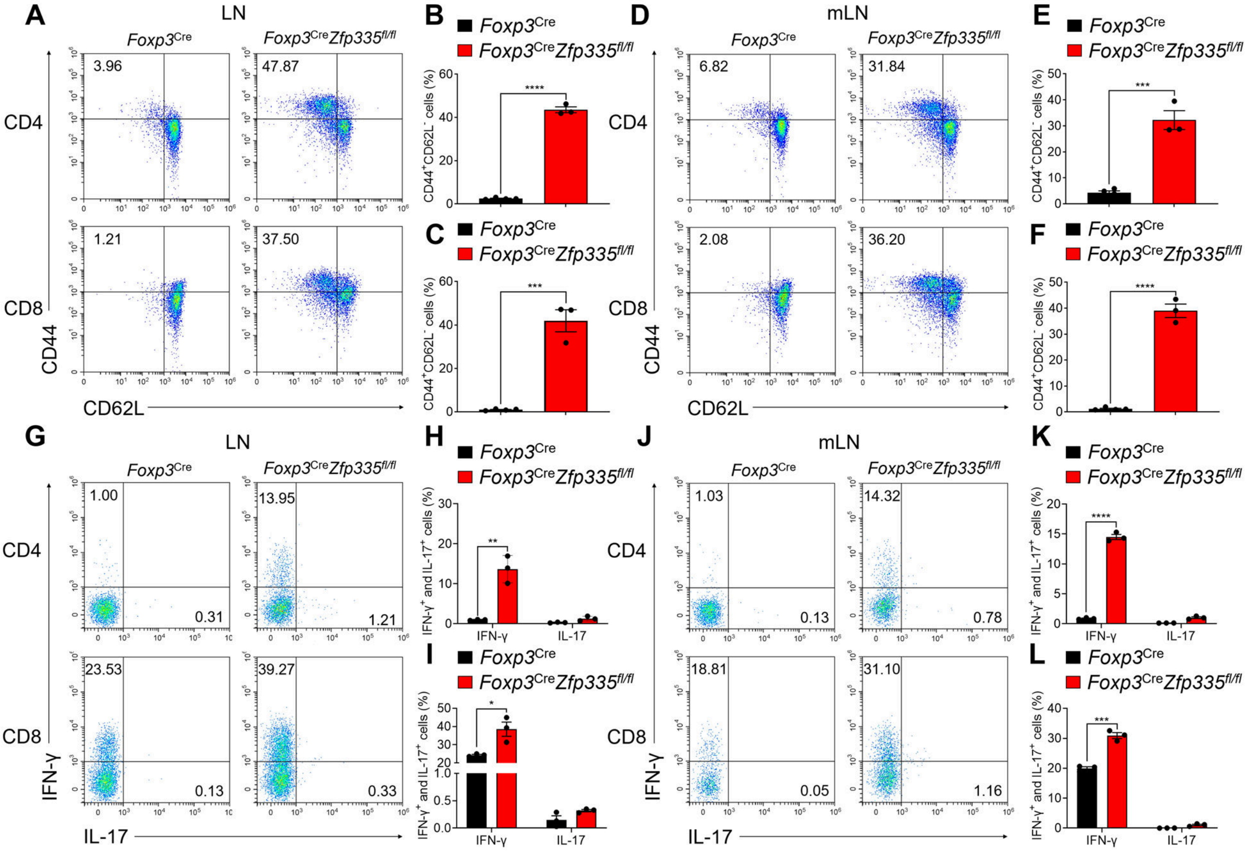

Supplemental Figure 5. Effector T cell analysis in *Foxp3CreZfp335fl/fl* mice. (**A**) Representative FACS plots of CD44 and CD62L expression in CD4+ and CD8+ T cells from LN of 3-week-old WT and KO mice. (**B** and **C**) Statistical frequencies of CD44+CD62L- cells in CD4+ (**B**) and CD8+ (**C**) T cells from LN (*n* = 3~4). (**D**) Representative FACS plots of CD44 and CD62L expression in CD4+ and CD8+ T cells from mLN of 3-week-old WT and KO mice. (**E** and **F**) Statistical frequencies of CD44+CD62L- cells in CD4+ (**E**) and CD8+ (**F**) T cells from mLN (*n* = 3~4). (**G**) Representative FACS plots of IFN-γ and IL-17 expression in CD4+ and CD8+ T cells from LN of 3-week-old WT and KO mice. (**H** and **I**) Statistical frequencies of IFN-γ+ and IL-17+ cells in CD4+ (**H**) and CD8+ (**I**) T cells from LN (*n* = 3). (**J**) Representative FACS plots of IFN-γ and IL-17 expression in CD4+ and CD8+ T cells from mLN of 3-week-old WT and KO mice. (**K** and **L**) Statistical frequencies of IFN-γ+ and IL-17+ cells in CD4+ (**K**) and CD8+ (**L**) T cells from mLN (*n* = 3). Data are representative of three independent experiments shown as the mean ± s.e.m. Statistical analysis is depicted as two-sided, unpaired *t* test; $^{*}P \le 0.05$, $^{**}P \le 0.01$, $^{***}P \le 0.001$, $^{****}P \le 0.0001$.

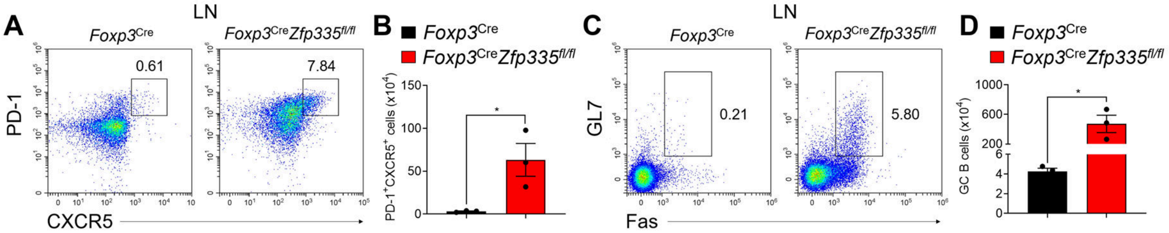

Supplemental Figure 6. Analysis of germinal center in the LN of *Foxp3*^{Cre}*Zfp335*$^{fl/fl}$ mice. (**A**) Representative FACS plots of PD-1 and CXCR5 expression in CD4$^+$ T cells from LN. (**B**) Cell number of PD-1$^+$CXCR5$^+$ cells in CD4$^+$ T cells from LN (n = 3). (**C**) Representative FACS plots of GL7 and Fas expression in CD19$^+$ B cells from LN. (**D**) Cell number of GL7$^+$Fas$^+$ (GC B) cells (n = 3). Data are representative of three independent experiments shown as the mean ± s.e.m. Statistical analysis is depicted as two-sided, unpaired t test; *$P \leq 0.05$.

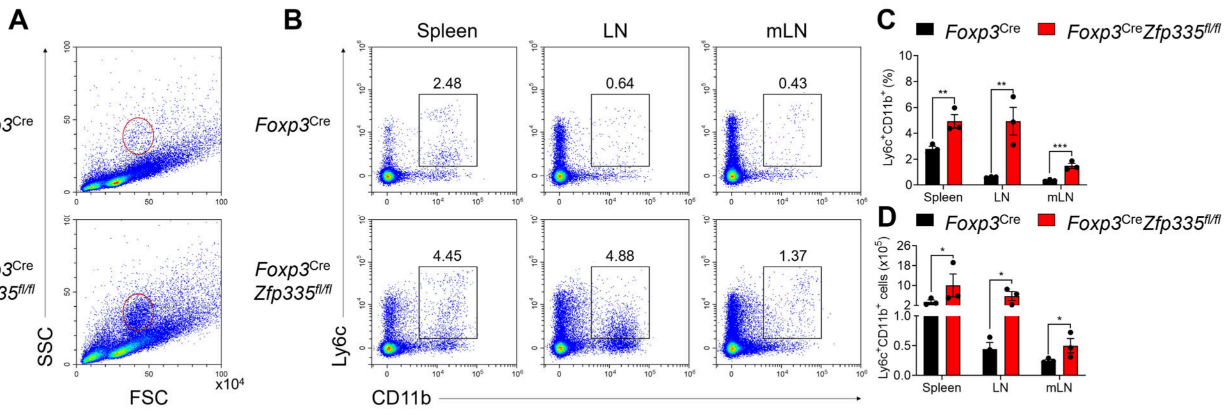

Supplemental Figure 7. Increased proportion of myeloid cells in *Foxp3^{Cre}Zfp335$^{fl/fl}$* mice. (**A**) Representative FACS plots of FSC and SSC with gating strategy in splenocytes of 3-week-old WT and KO mice. (**B**) Representative FACS plots of Ly6c and CD11b expression in cells from spleen, LN and mLN. (**C** and **D**) Statistical frequencies (**C**) and numbers (**D**) of Ly6c$^+$ CD11b$^+$ cells (*n* = 3). Data are representative of three independent experiments shown as the mean ± s.e.m. Statistical analysis is depicted as two-sided, unpaired *t* test; *$P \leq 0.05$, **$P \leq 0.01$, ***$P \leq 0.001$.

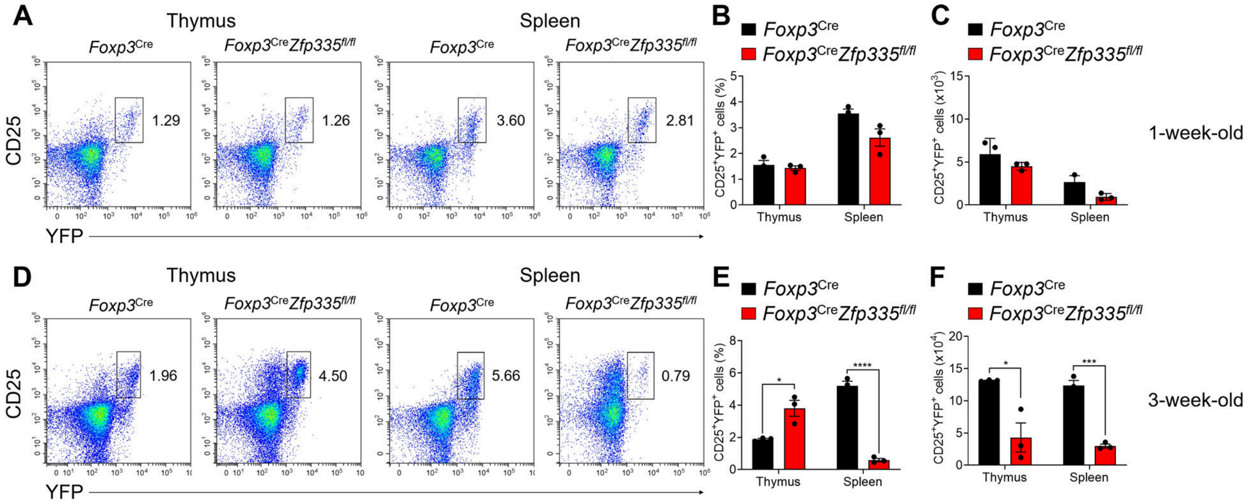

Supplemental Figure 8. Effect of Zfp335 deletion on T_{reg} cell population in the thymus and spleen. (**A**) Representative FACS plots of $CD25^{+}YFP^{+}$ T_{reg} cells in the thymus and spleen of 1-week-old WT and KO mice. (**B** and **C**) Statistical frequencies (**B**) and numbers (**C**) of $CD25^{+}YFP^{+}$ T_{reg} cells in the thymus and spleen (n = 3). (**D**) Representative FACS plots of $CD25^{+}YFP^{+}$ T_{reg} cells in the thymus and spleen of 3-week-old WT and KO mice. (**E** and **F**) Statistical frequencies (**E**) and numbers (**F**) of $CD25^{+}YFP^{+}$ T_{reg} cells in the thymus and spleen (n = 3). Data are representative of three independent experiments shown as the mean ± s.e.m. Statistical analysis is depicted as two-sided, unpaired t test; *$P \leq 0.05$, ***$P \leq 0.001$, ****$P \leq 0.0001$.

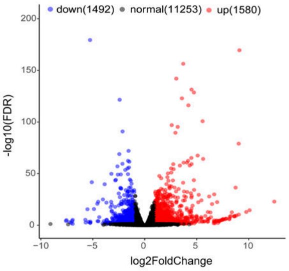

Supplemental Figure 9. Volcano plot of Zfp335-sufficient and -deficient T_{reg} cells. Volcano plot showing the gene signature of *Foxp3*^{Cre} and *Foxp3*^{Cre}*Zfp335*$^{fl/fl}$ T_{reg} cells. X-axis represents log2-transformed fold change. Y-axis represents −log10 transformed significance. Red points represent up-regulated DEGs. Blue points represent down-regulated DEGs.

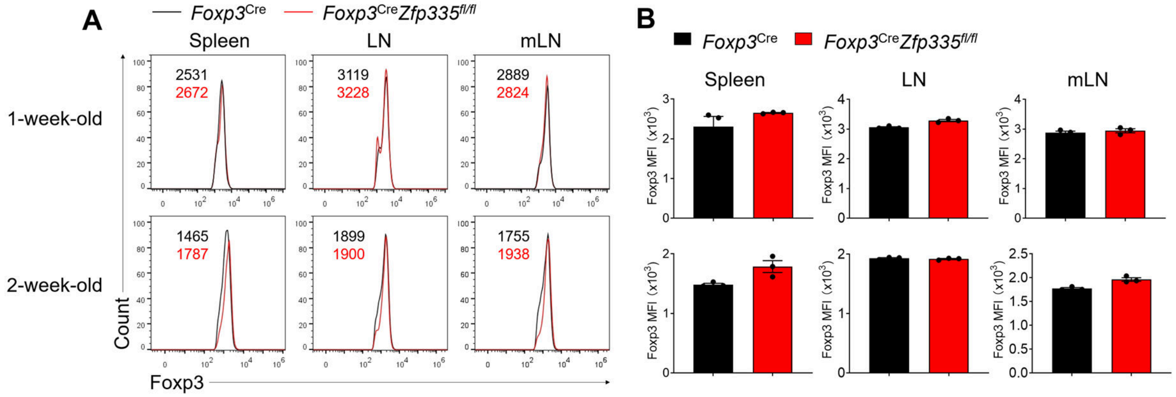

Supplemental Figure 10. Foxp3 protein expression in Zfp335-deficient T_{reg} cells. (**A**) Representative histogram of Foxp3 expression in T_{reg} cells from spleen, LN and mLN of WT and KO (*Foxp3^{Cre}Zfp335$^{fl/fl}$*) mice at 1- and 2-week age. (**B**) MFI of Foxp3 in (**a**) (*n* = 3). Data are representative of three independent experiments shown as the mean ± s.e.m. Statistical analysis is depicted as two-sided, unpaired *t* test.

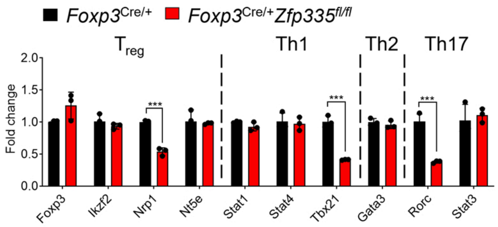

Supplemental Figure 11. Expression of genes related with different Th lineages in heterozygous *Foxp3*$^{Cre/+}$ZFP335$^{f/f}$ female mice. Relative changes of mRNA expression of genes associated with T_{reg}, Th1, Th2 and Th17 lineage signature in CD4^{+}CD25^{+}YFP^{+} T_{reg} cells from *Foxp3*$^{Cre/+}$ control and heterozygous *Foxp3*$^{Cre/+}$*Zfp335*$^{fl/fl}$ female mice (n = 3). Data are representative of three independent experiments shown as the mean ± s.e.m. Statistical analysis is depicted as two-sided, unpaired t test.

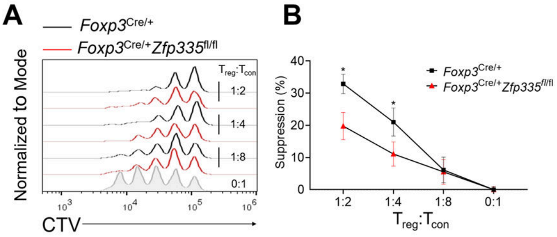

Supplemental Figure 12. Zfp335 deficiency impairs suppressive function of T_{reg} cells in vitro. (**A**) Representative histogram of CellTrace Violet (CTV) dilution of T_{con} cells. Naïve $CD4^+$ T cells were stimulated with anti-mCD3 Ab and APC cells in the presence of T_{reg} cells from *Foxp3*$^{Cre/+}$ or *Foxp3*$^{Cre/+}$*Zfp335*$^{fl/fl}$ mice for 60h. The different ratios of T_{reg} vs T_{con} cells were included. (**B**) Percentage of suppression by T_{reg} cells (n = 3). Data are representative of two independent experiments shown as the mean ± s.e.m. Statistical analysis is depicted as two-sided, unpaired t test; $^*P \leq 0.05$.

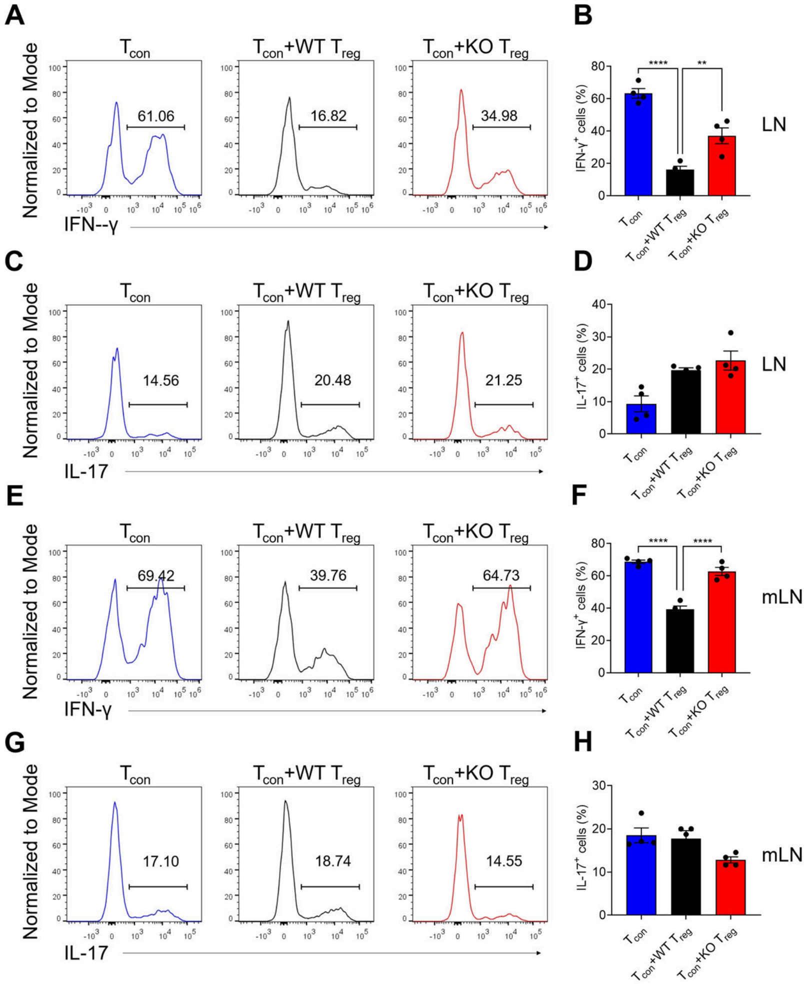

Supplemental Figure 13. Impaired suppressive function of Zfp335 deficient T_{reg} cells in colitis model. Colitis model induced by adoptive transfer of wild-type naïve ($CD4^+CD25^-CD45RB^{hi}$) T cells together with either PBS (T_{con}, *n* = 4) or sorted T_{reg} cells from 6-week-old wild-type mice (ER^{Cre}; T_{con} + WT T_{reg}, *n* = 4) or Zfp335 knock-out mice (ER^{Cre}*Zfp335*$^{fl/fl}$;T_{con} + KO T_{reg}, *n* = 4). (**A**-**D**) Representative FACS plots of IFN-γ (**A**) and IL-17 (**C**) expression in $CD4^+$ T cells from LN. Statistical frequencies of IFN-$γ^+$ (**B**) and IL-17^+ (**D**) cells in $CD4^+$ T cells from LN. (**E**-**H**) Representative FACS plots of IFN-γ (**E**) and IL-17 (**G**) expression in $CD4^+$ T cells from mLN. Statistical frequencies of IFN-$γ^+$ (**F**) and IL-17^+ (**H**) cells in $CD4^+$ T cells from mLN. Data are representative of two independent experiments shown as the mean ± s.e.m. Statistical analysis is depicted as 1-way ANOVA with Tukey's multiple-comparison test; $^{**}P \leq 0.01$, $^{****}P \leq 0.0001$.

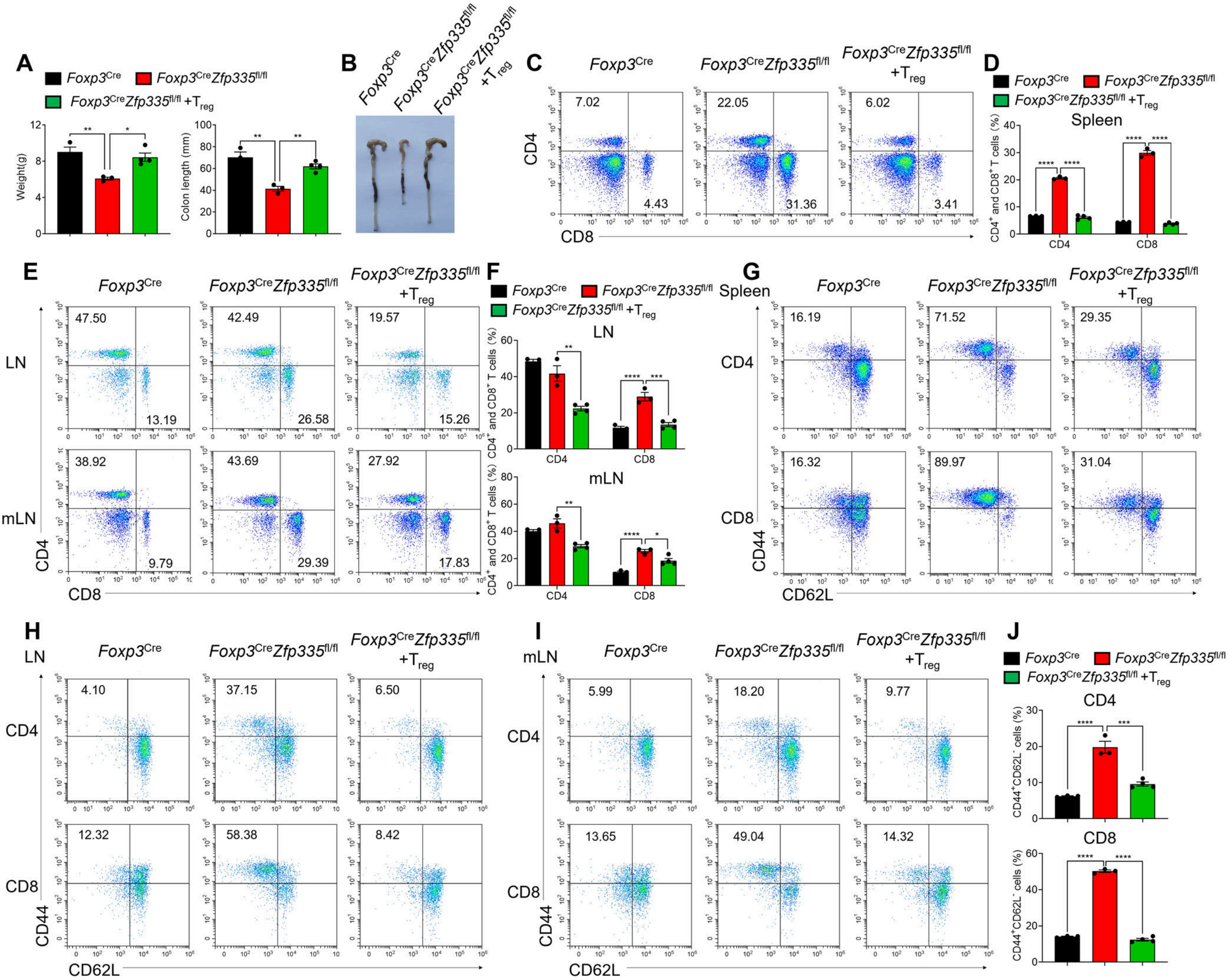

Supplemental Figure 14. Compromised immunosuppressive ability of Zfp335-deficient T_{reg} cells. (**A**) Body weight (Left) and Colon length (Right) of WT (*n* = 3), KO (*n* = 3) and KO mice transferred with WT (CD45.1⁺) T_{reg} cells (KO+T_{reg}) at 3-week-old (*n* = 4). (**B**) Representative images of colons from WT, KO and KO+ T_{reg} mice. (**C**) Representative FACS plots of CD4 and CD8 expression in splenocytes from WT, KO and KO+ T_{reg} mice. (**D**) Statistical frequencies of splenic $CD4^+$ and $CD8^+$ T cells (*n* = 3~4). (**E**) Representative FACS plots of CD4 and CD8 expression in LN and mLN from WT, KO and KO+T_{reg} mice. (**F**) Statistical frequencies of $CD4^+$ and $CD8^+$ T cells in LN (Up) and mLN (Down) (*n* = 3~4). (**G-I**) Representative FACS plots of CD44 and CD62L expression in $CD4^+$ and $CD8^+$ T cells from the spleen (**G**), LN (**H**) and mLN (**I**) of WT, KO and KO+T_{reg} mice. (**J**) Statistical frequencies of $CD44^+CD62L^-$ cells in $CD4^+$ T cells from mLN of WT, KO and KO+T_{reg} mice (*n* = 3~4). Data are representative of two independent experiments shown as the mean ± s.e.m. Statistical analysis is depicted as 1-way ANOVA with Tukey's multiple-comparison test; $^*P \le 0.05$, $^{**}P \le 0.01$, $^{***}P \le 0.001$, $^{****}P \le 0.0001$.

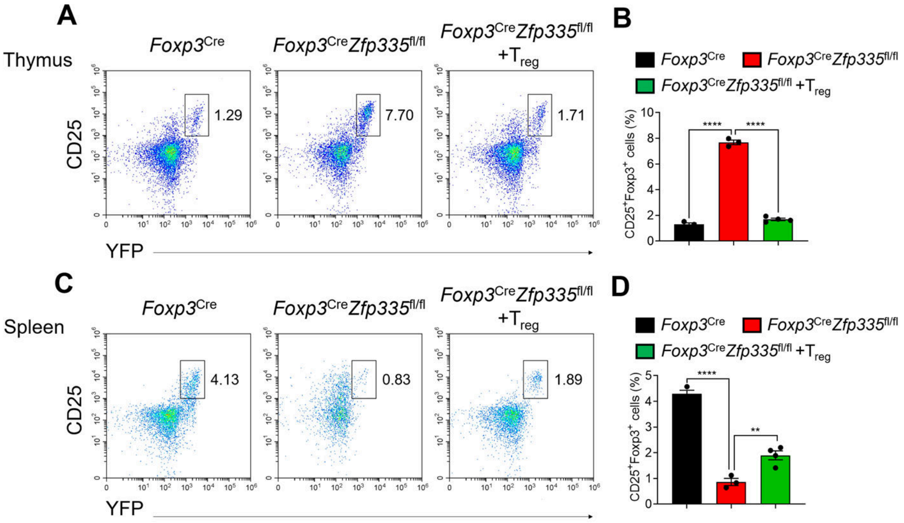

Supplemental Figure 15. Restoration of Zfp335-deficient T_{reg} cells in the presence of WT T_{reg} cells. (**A**) Representative FACS plots of CD25 and YFP expression among $CD4^+CD8^-$ thymocytes from WT, KO and KO+T_{reg} mice. (**B**) Statistical frequencies of $CD25^+YFP^+$ cells (*n* = 3~4). (**C**) Representative FACS plots of CD25 and YFP expression in splenic $CD4^+$ T cells from WT, KO and KO+T_{reg} mice. (**D**) Statistical frequencies of $CD25^+YFP^+$ cells (*n* = 3~4). Data are representative of two independent experiments shown as the mean ± s.e.m. Statistical analysis is depicted as 1-way ANOVA with Tukey's multiple-comparison test; $^{**}P \leq 0.01$, $^{****}P \leq 0.0001$.

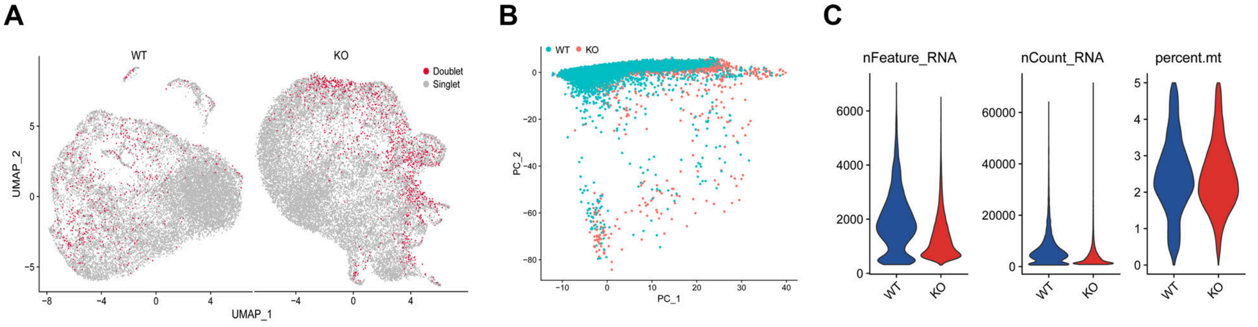

Supplemental Figure 16. Quality control and normalization of scRNA-seq data. (**A**) UMAP projections of doublets in WT mice (Left) and KO mice (Right). (**B**) PCA plot of high-quality T_{reg} cells showing no batch effect between WT and KO mice, colors coded by groups. (**C**) Violin plots showing the data quality after filtering out the cells with poor quality.

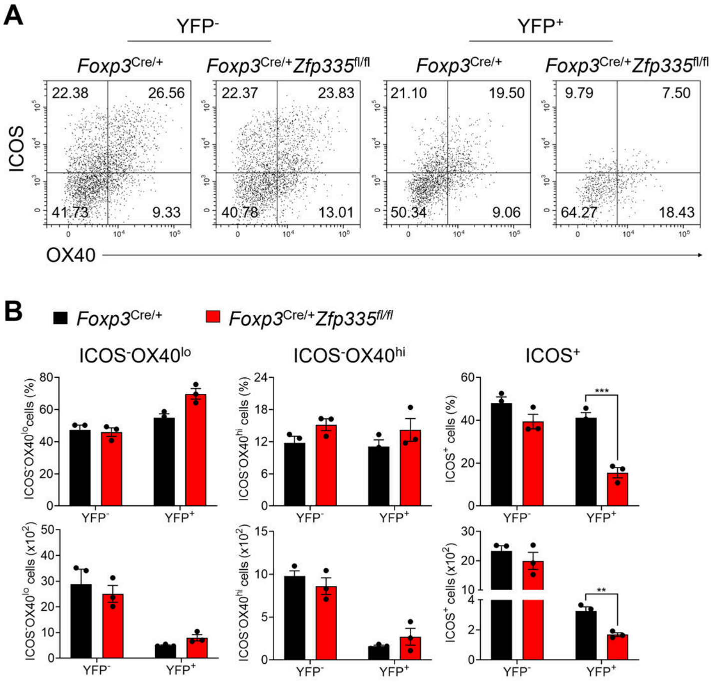

Supplemental Figure 17. Effect of Zfp335 deletion on T_{reg} subsets in control and heterozygous *Foxp3*^Cre/+^*Zfp335*^fl/fl^ female mice. (**A**) Representative FACS plots of ICOS and OX40 expression in T_{reg} cells from *Foxp3*$^{Cre/+}$ and *Foxp3*$^{Cre/+}$*Zfp335*$^{fl/fl}$ female mice. (**B**) Statistical frequencies (Up) and number (Down) of ICOS$^-$OX40^{lo}, ICOS$^-$OX40^{hi} and ICOS$^+$ cells for (**A**) (n = 3). Data are representative of two independent experiments shown as the mean ± s.e.m. Statistical analysis is depicted as two-sided, unpaired *t* test; $^{**}P \leq 0.01$, $^{***}P \leq 0.001$.

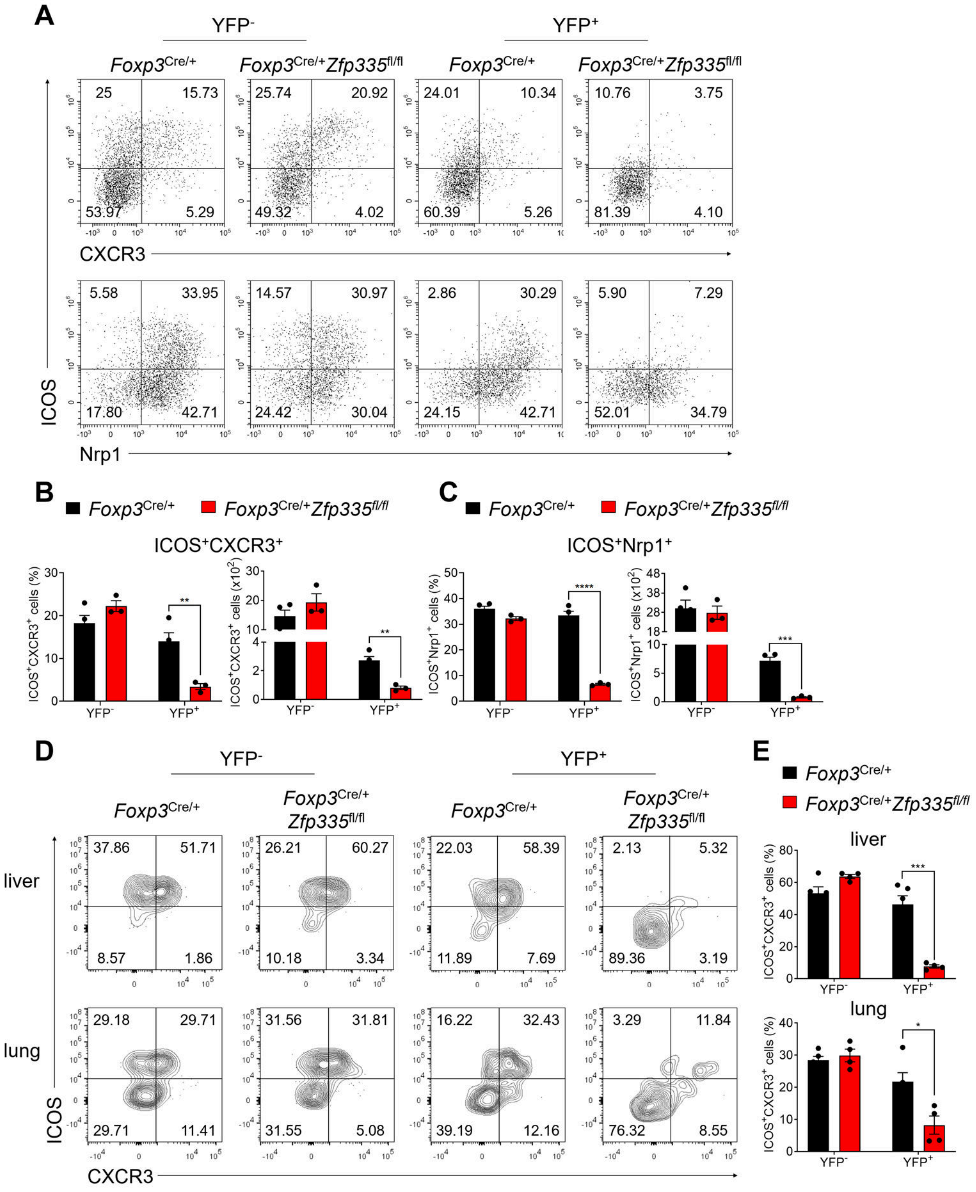

Supplemental Figure 18. Zfp335 deletion leads to reduced eT$_{reg}$ cells in control and heterozygous *Foxp3*$^{Cre/+}$*Zfp335*$^{fl/fl}$ female mice. (**A**) Representative FACS plots of ICOS, CXCR3 and Nrp1 expression in T$_{reg}$ cells from *Foxp3*$^{Cre/+}$ and *Foxp3*$^{Cre/+}$*Zfp335*$^{fl/fl}$ female mice. (**B**) Statistical frequencies of ICOS$^+$CXCR3$^+$ cells for (**A**) (n = 3~4). (**C**) Statistical frequencies of ICOS$^+$Nrp1$^+$ cells for (**A**) (n = 3~4). (**D**) Representative FACS plots of ICOS and CXCR3 expression in T$_{reg}$ cells from liver and lung of *Foxp3*$^{Cre/+}$ and *Foxp3*$^{Cre/+}$*Zfp335*$^{fl/fl}$ female mice. (**E**) Statistical frequencies of ICOS$^+$CXCR3$^+$ cells for (**D**) (n = 4~5). Data are representative of two independent experiments shown as the mean ± s.e.m. Statistical analysis is depicted as two-sided, unpaired t test; *$P \leq 0.05$, **$P \leq 0.01$, ***$P \leq 0.001$, ****$P \leq 0.0001$.

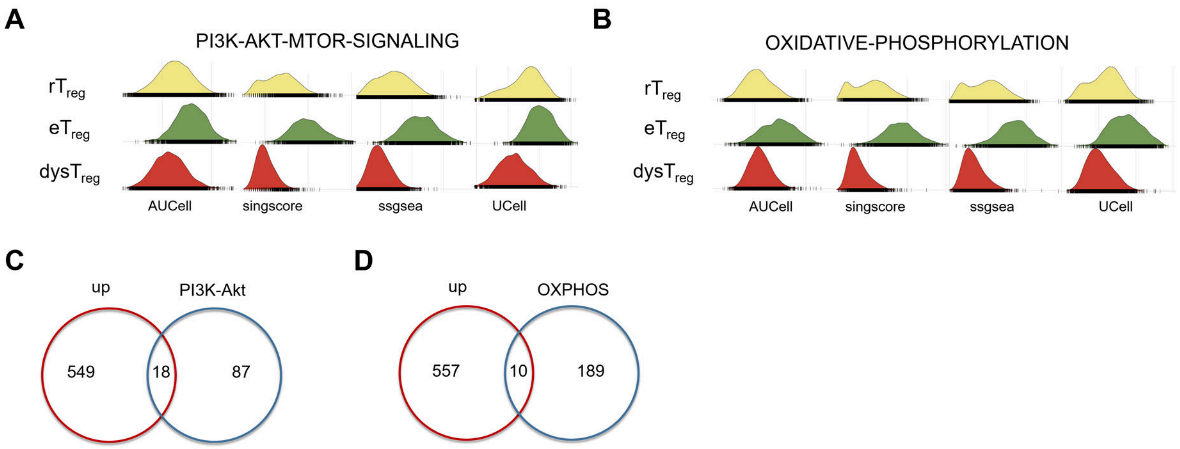

Supplemental Figure 19. Identification of genes associated with metabolism pathways. (**A**) Ridgeplots showing the PI3K-AKT-MTOR-SIGNALING scores in rT_{reg}, $dysT_{reg}$ and eT_{reg} based on AUCell, singscore, ssgsea and Ucell methods. (**B**) Ridgeplots showing the OXIDATIVE-PHOSPHORYLATION scores in rT_{reg}, $dysT_{reg}$ and eT_{reg} based on AUCell, singscore, ssgsea and Ucell methods. (**C**) Venn diagram shows the genes shared between upregulated genes (eT_{reg} vs $dysT_{reg}$) and genes in PI3K-AKT-MTOR-SIGNALING set. (**D**) Venn diagram shows the genes shared between upregulated genes (eT_{reg} vs $dysT_{reg}$) and genes in OXIDATIVE-PHOSPHORYLATION set.

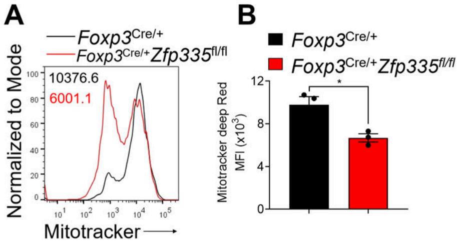

Supplemental Figure 20. Mitotracker mass of T_{reg} cells from control and heterozygous *Foxp3*$^{Cre/+}$*Zfp335*$^{fl/fl}$ female mice. (**A**) Representative FACS plots of Mitotracker deep Red staining in *Foxp3*$^{Cre/+}$ and *Foxp3*$^{Cre/+}$*Zfp335*$^{fl/fl}$ rT_{reg} cells activated with anti-mCD3/CD28 Abs and IL-2 for 3 days. (**B**) The statistics of Mitotracker deep Red MFI in (**A**) (*n* = 3). Data are representative of two independent experiments shown as the mean ± s.e.m. Statistical analysis is depicted as two-sided, unpaired t-tests; $^*P \leq 0.05$.

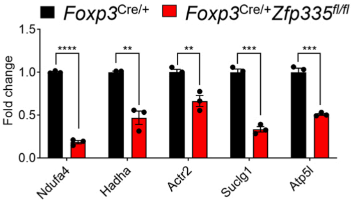

Supplemental Figure 21. Expression of overlapping genes in T_{reg} cells of control and heterozygous *Foxp3*$^{Cre/+}$*Zfp335*$^{fl/fl}$ female mice. The relative changes of mRNA expression of overlapping genes in $CD4^+CD25^+YFP^+$ T_{reg} cells from *Foxp3*$^{Cre/+}$ and *Foxp3*$^{Cre/+}$*Zfp335*$^{fl/fl}$ female mice (n = 3). Data are representative of three independent experiments shown as the mean ± s.e.m. Statistical analysis is depicted as two-sided, unpaired t test; $^{**}P \leq 0.01$, $^{***}P \leq 0.001$, $^{****}P \leq 0.0001$.

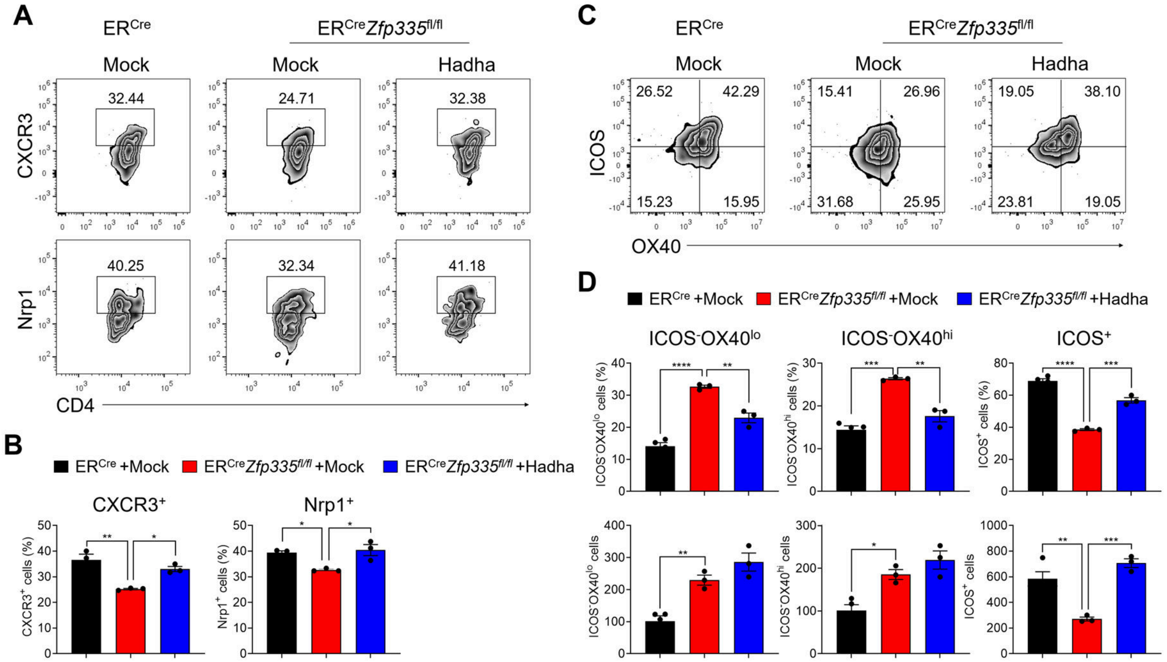

Supplemental Figure 22. Effects of Hadha overexpression on T_{reg} subsets. (**A**) Representative FACS plots of CXCR3 and Nrp1 expression in T_{reg} cells transfected with Mock and *Hadha.* (**B**) Statistical frequencies of CXCR3⁺ and Nrp1⁺ T_{reg} cells in different groups for (**A**) (*n* = 3). (**C**) Representative FACS plots of ICOS and OX40 expression in T_{reg} cells transfected with Mock and *Hadha.* (**D**) Statistical frequencies of $ICOS^-OX40^{lo}$, $ICOS^-OX40^{hi}$ and $ICOS^+$ T_{reg} cells in different groups for (**C**) (*n* = 3~4). Data are representative of two independent experiments shown as the mean ± s.e.m. Statistical analysis is depicted as 1-way ANOVA with Tukey's multiple-comparison test; **P* ≤ 0.05, ***P* ≤ 0.01, ****P* ≤ 0.001, *****P* ≤ 0.0001.

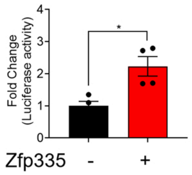

Supplemental Figure 23. Validation of Zfp335 and *Hadha* binding. Luciferase activity in 293T cell lysate following transfection of the Luci_Hadha_promoter with Mock or Zfp335 expression vector (n = 4). Data are representative of three independent experiments shown as the mean ± s.e.m. Statistical analysis is depicted as two-sided, unpaired t test; *$P \leq 0.05$.

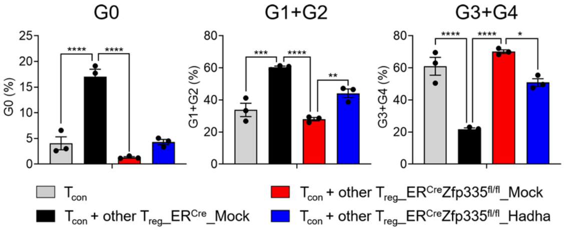

Supplemental Figure 24. Effect of Zfp335 deletion on the suppressive function of other T_{reg} cells. Statistical frequencies of each cell division of Tcon cells in the presence of WT other T_{reg} cells transfected with Mock and KO other T_{reg} cells transfected with Mock or Hadha (*n* = 3). Data are representative of three independent experiments shown as the mean ± s.e.m. Statistical analysis is depicted as 1-way ANOVA with Tukey's multiple-comparison test; **P* ≤ 0.05, ***P* ≤ 0.01, ****P* ≤ 0.001, *****P* ≤ 0.0001.

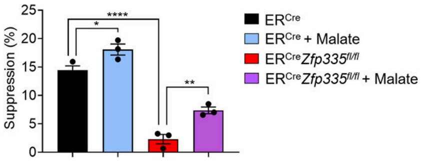

Supplemental Figure 25. Effects of malate treatment on T_{reg} function. Percentage of suppression by T_{reg} cells from ER^{Cre} and ER^{Cre}*Zfp335*$^{fl/fl}$ mice with or without malate treatment (n = 3). Data are representative of two independent experiments shown as the mean ± s.e.m. Statistical analysis is depicted as 1-way ANOVA with Tukey's multiple-comparison test; *$P \leq 0.05$, **$P \leq 0.01$, ****$P \leq 0.0001$.

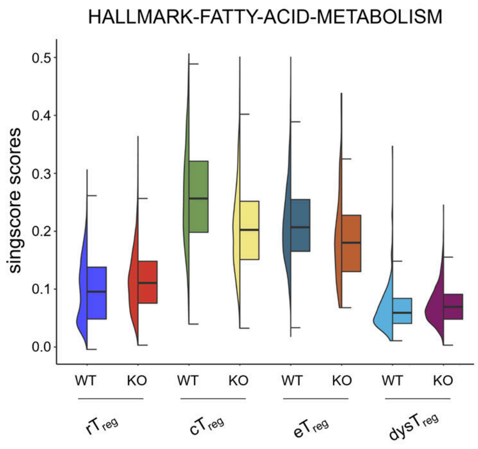

Supplemental Figure 26. Halfvlnplot of fatty acid metabolism pathway. Halfvlnplot showing FATTY−ACID−METABOLISM pathway score in different T_{reg} subsets based on singscore method.

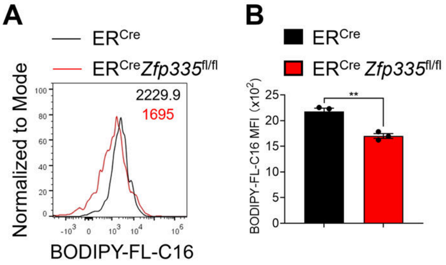

Supplemental Figure 27. Fatty acid uptake of Zfp335-deficient ICOS$^+$ eT$_{reg}$ cells. (**A**) Fatty acid uptake was examined by BODIPY-FL-C16 and representative FACS plots of BODIPY-FL-C16 incorporation in ICOS$^+$ T$_{reg}$ cells from ER^{Cre} and ER^{Cre}*Zfp335*$^{fl/fl}$ mice. (**B**) MFI of BODIPY-FL-C16 in ICOS$^+$ T$_{reg}$ cells from ER^{Cre} and ER^{Cre}*Zfp335*$^{fl/fl}$ mice (n = 3). Data are representative of three independent experiments shown as the mean ± s.e.m. Statistical analysis is depicted as two-sided, unpaired t test; **$P \leq 0.01$.

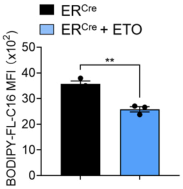

Supplemental Figure 28. Fatty acid uptake of 40 μM etomoxir-treated T_{reg} cells. Fatty acid uptake was examined by BODIPY-FL-C16 and MFI of BODIPY-FL-C16 in T_{reg} cells after 40 μM etomoxir treatment (*n* = 3). Data are representative of three independent experiments shown as the mean ± s.e.m. Statistical analysis is depicted as two-sided, unpaired *t* test; $^{**}P \leq 0.01$.

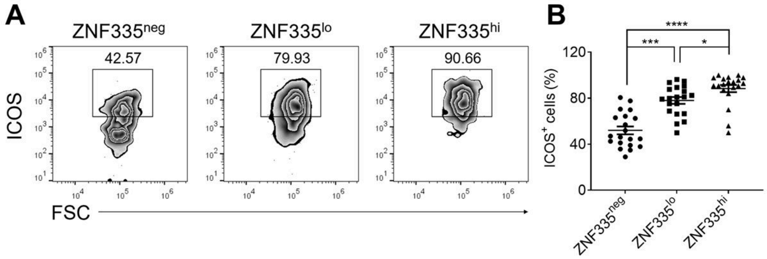

Supplemental Figure 29. Correlative analysis between ZNF335 expression and ICOS+ T_{reg} cells. (**A**) Representative FACS plots of ICOS+ T_{reg} cells in ZNF335^{neg}, ZNF335^{lo} and ZNF335^{hi} T_{reg} cells of HD. (**B**) Statistical frequencies of ICOS+ T_{reg} cells for (**A**) (n = 20). Data are shown as the mean ± s.e.m. Statistical analysis is depicted as Kruskal-Wallist test with two-stage step-up procedure of Benjamini, Krieger and Yekutieli multiple-comparison test; *$P \leq 0.05$, ***$P \leq 0.001$, ****$P \leq 0.0001$.

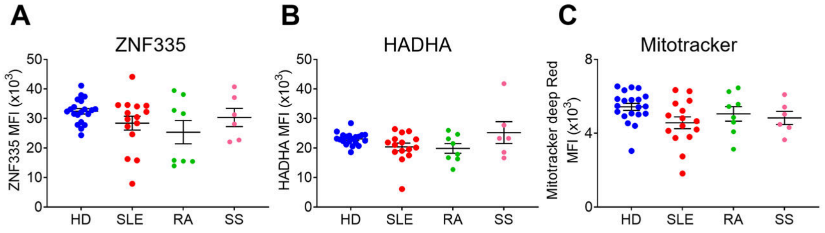

Supplemental Figure 30. The expression of ZNF335, HADHA and Mitotracker in CD4$^+$ T$_{con}$ cells upon TCR and IL-2 stimulation. MFI of ZNF335 (**A**), HADHA (**B**) and Mitotracker deep Red (**C**) in HD (*n* = 20) and patients with SLE (*n* = 15), RA (*n* = 8) and SS (*n* = 6). Data are shown as the mean ± s.e.m. Statistical analysis is depicted as Kruskal-Wallist test with two-stage step-up procedure of Benjamini, Krieger and Yekutieli multiple-comparison test.
